# Can past variants of SARS-CoV-2 predict the impact of future variants? Machine learning for early warning of US counties at risk

**DOI:** 10.1007/s10729-025-09728-4

**Published:** 2025-10-13

**Authors:** Kevin B. Smith, Siqian Shen, Brian T. Denton

**Affiliations:** Industrial and Operations Engineering, University of Michigan, Ann Arbor, MI USA

**Keywords:** Pandemic preparedness, County-level analysis, Machine learning algorithms, Stochastic Gradient Boosted Average Precision (SGBAP), SARS-CoV-2

## Abstract

**Abstract:**

In this paper, we determine whether machine learning (ML) models created using data from the novel SARS-CoV-2 Alpha variant can prospectively predict county-level incidence of emerging variants, validated using data of the Omicron variant. We first select publicly-available sociodemographic, economic, and health-related characteristics of 3140 United States (US) counties at the time of the confirmed early US outbreak of the novel SARS-CoV-2 virus in March 2020 for analysis. Our primary result is the set of US counties that experienced the upper quartile of population-adjusted Omicron variant incidence at certain period (e.g., 100 days) after Omicron variant’s appearance in the US. We show more predictive results by incorporating additional data and features (e.g., human mobility) that can be acquired dynamically after the outbreak, to improve prediction accuracy at the cost of additional waiting time and effort. Towards the goal of decision support, we aim to prospectively evaluate our models’ ability to classify and rank US counties at risk. We measure their classification performance using the area under receiver operating characteristic curve score with 95% confidence intervals. We further calculate the proportion of the top counties by Omicron incidence that our models correctly identify, and compare their score with those of individual county-level features that can serve as a heuristic predictive performance baseline. Our results show that ML algorithms predict county-level Omicron variant incidence with better performance than natural heuristics that decision makers might otherwise use. More generally, historical data from the first wave of a novel pandemic can help predict the incidence of future variants and strengthen state or federal pandemic response interventions.

**Highlights:**

- Data-driven predictive models that capture patterns from early viral variants can support policymaking related to emerging viral variants.
- County-level sociodemographic, health, and economic characteristics are predictive of early COVID-19 outcomes in the United States (US).
- Machine learning models trained on early US county-level COVID-19 outcomes are additionally predictive of county-level SARS-CoV-2 Omicron variant outcomes.
- County-level machine learning models can be used as a critical policymaking tool given the inevitability of novel emerging viruses.

**Supplementary Information:**

The online version contains supplementary material available at 10.1007/s10729-025-09728-4.

## Introduction

Health systems in the United States (US) have been cited as the weakest area of national pandemic preparedness [54]. Improvements could be achieved through centralized and evidence-based assignment of scarce critical medical resources [48] if pandemic burden can be predicted in advance [53]. Moreover, data-driven classification models based on Machine Learning (ML) algorithms have helped US public health policymakers make county-level resource allocation decisions to contain outbreaks of a novel virus or its variants [see, e.g., 73].

In response to early indications of a pandemic outbreak of severe acute respiratory syndrome coronavirus 2 (SARS-CoV-2) in 2020, decision makers at the federal, state, and local levels had to allocate scarce resources, like ventilators and clinical personnel, regionally across the US. These decisions were often made in response to coronavirus disease 2019 (COVID-19) health outcomes that lag the spread of the underlying SARS-CoV-2 virus. The largest opportunity to control outbreaks and, subsequently, pandemics occurs early in the respective cycle of the spread of disease [2], most notably when considering the impact of contact tracing and case isolation [20]. A coordinated health response could reduce disparities caused by pandemic burden. However, public health decision makers need advance knowledge of regions most likely to experience high incidence at the onset of an outbreak [19]. To the best of our knowledge, no previous literature has shown whether county-level burden of a novel virus or its variants can be predicted in advance without using epidemic data from the current dominant viral variant.

To address this knowledge gap, we apply ML (specifically, Stepwise Logistic Regression (LR), and Stochastic Gradient Boosted Average Precision (SGBAP)) to baseline county-level data and data for the Alpha variant of the SARS-CoV-2 virus. Our baseline county-level data included population-level demographic, economic factors, transportation networks, and health-related features for US counties. We prospectively validate the algorithms’ potential to predict outcomes in advance, for the emerging SARS-CoV-2 Omicron variant. We compare the performance of the models to discriminate and rank which US counties may experience the highest pandemic burden before the emergence of the novel Omicron variant with the performance of two features that we propose that a policymaker may utilize heuristically. In particular, we sort those two features, a county’s 1) population density and 2) cumulative reported COVID-19 cases per 100k population on April 30, 2020, in decreasing order to simulate an approach for decision makers to determine the counties most at-need (i.e., the counties most at-need could be determined as those at the top of a list sorted on a feature’s values in decreasing order) in advance of an emerging SARS-CoV-2 variant like Omicron. Furthermore, our approach can associate the impact of county-level factors that serve to classify the burden experienced at the onset of emerging variants.

### Main contributions

This article contributes to the related literature above as follows. First, we develop, to our knowledge, the first ML framework that predicts, in advance, the US counties at risk of burden attributable to emerging variants of SARS-CoV-2 and thus close a critical knowledge gap about whether county-level burden of a novel virus or its variants can be predicted in advance without detailed epidemiological data. We validate the results using real data and outcomes from Alpha and Omicron variants in the US. Second, we further include dynamic data and features such as mobility data, in addition to demographic data, to simulate a dynamic predictive model to improve prediction accuracy. Third, we demonstrate how our framework could become an important element of a US spatial decision support system for pandemic prevention, thus enabling policymakers to generate interventions proactively and leading to earlier pandemic control than was possible with early SARS-CoV-2 interventions.

The rest of our paper is organized as follows. In Section 2, we conduct a comprehensive literature review and outline several streams of research related to predicting county-level SARS-CoV-2. In Section 3, we extract publicly-accessible data to develop features for our ML algorithms, and then explicitly define the county-level outcomes that our models will be trained to predict. In Section 4, we introduce the ML algorithms used in our study and visualize the flow of our analytic approach. In Section 5, we present the classification and ranking results of our ML models and compare them with heuristic baselines to demonstrate the value of a data-driven approach. We conclude the paper in Section 6 and also present future research directions to address the current limitations.

## Related work

We outline several streams of research related to predicting SARS-CoV-2 at the county-level. We first review a variety of studies that have applied ML to the prediction of SARS-CoV-2 at the county level and identify several types of outcomes published previously. We then summarize works that describe the pitfalls in leveraging SARS-CoV-2 data within an epidemiological surveillance system. We summarize interpretable predictive approaches to the county-level prediction problem and then review examples of how data-driven models can be used as decision support tools.

### Regional SARS-CoV-2 prediction

Lucas et al. [45] applied deep learning approaches to conduct spatiotemporal incidence forecasting in the US while Sciannameo et al. [62] applied deep learning for regional forecasts in Italy. Furthermore, Price et al. [57] applied deep learning to aid decisions about which regional locations should be prioritized for increased testing according to epidemiological forecasts and only in the state of West Virginia.

### SARS-CoV-2 outcomes

Other studies have applied ML to forecast morbidity and mortality [see, e.g., 27, 34] as well as values and trends in viral incidence [see 52, 70, 74]. ML algorithms have also been applied to predict daily trends [42], vaccination levels [1], and pandemic vulnerability [47, 67] at the county level.

### SARS-CoV-2 data pitfalls

In this study, we propose to develop a tool that would serve to inform resource allocation decisions as an important element part of an epidemiological surveillance system. We now review the literature on possible pitfalls of including epidemiological data in the development of such a tool.

Several attributes have been defined to help evaluate the effectiveness of an epidemiological surveillance system. The two we will discuss in this context are timeliness and representativeness. The timeliness attribute has been defined as the time gap between steps in the surveillance system whereas a system’s representativeness is represented as the accuracy of disease occurrences that are reported over time and the distribution of those occurrences regionally [see 40]. An evaluation timeliness of epidemiological surveillance systems for a variety of infectious diseases has been conducted by Jajosky and Groseclose [35], while Bansal et al. [4] define a knowledge gap related to the representativeness in ’big data’ streams for epidemiological surveillance. There have also been attempts to develop modeling frameworks that correct for reporting delays in epidemiological data, thus purporting to improve timeliness of epidemiological systems including Bastos et al. [5] and Sarnaglia et al. [61].

Surveilling SARS-CoV-2 accurately requires that the underlying data used exhibit timeliness and representativeness. That is, the underlying SARS-CoV-2 data must exhibit a stable time gap and accurately represent the true disease incidence regionally. However, it has been well-studied that these data exhibit numerous problems, thus limiting their application for decision making [see 23]. Badker et al. [3] also reviewed SARS-CoV-2 data challenges. In particular, they describe how a variety of COVID-19 data sources emerged throughout the pandemic but without a standardization plan. They further explained when reporting epidemic data, several organization experienced variable time gaps and lacked a standard case definition. Pearce et al. [55] reported that testing data from early in the SARS-CoV-2 pandemic was derived largely from patients with symptoms while regional differences in test performance were likely. Taken together, these challenges with SARS-CoV-2 data may lead to pitfalls such as uncertainty in risk prediction and inappropriate policy guidance.

### Interpretable SARS-CoV-2 prediction: Feature importance

In the context of the county-level prediction problem, most of the research related to interpretable ML models relied on assessing feature importance. ML approaches have been applied to assess county-level feature importance associated with disease incidence [14, 26, 74] and mortality [27, 68]. One particularly rich area for feature importance has been case rate associations with mobility metrics [32, 50, 77]. A study by Li et al. [43] conducted an estimate of feature importance for three unique stages of a single COVID-19 variant. The feature importance insights generated by Li et al. [43] could be useful in decision making if the models used to generate them exhibited good discriminative performance. However, without such an evaluation, it is not clear in what way the features contribute to the predictive task. In contrast to Li et al. [43], our approach allows for the direct assessment of the features involved in the county-level risk prediction problem on the predictive performance of the models we validate retrospectively and prospectively for emerging SARS-CoV-2 variants.

### SARS-CoV-2 prediction decision support

Research at the intersection of data-driven modeling and decision support using SARS-CoV-2 data has involved predicting outbreaks at correctional facilities [46] and developing a population-based vulnerability index [47]. In each of these studies, the authors developed data-driven models using SARS-CoV-2 data and then used their predictions to guide decision making. While these approaches closely mirror our approach, in this paper, we chose to avoid the limitations associated with the quality of SARS-CoV-2 data summarized above and thus exclude it from training our models.

In Queiroz et al. [58], the authors conducted a structured literature review to evaluate the impacts of epidemic outbreaks on commercial supply chains. They proposed the creation of epidemiological compartmental models to study structured effects on supply chains and thus, support decision making. In the same vein, Büyüktahtakın et al. [13] developed an optimization approach to model the effect of spatial epidemic spread on logistics systems and suggested that their framework could support policy recommendation. Decision-making and optimization models have been demonstrated for use in allocating scarce medical resources in the US [63] and Italy [25] while Bertsimas et al. [7] developed a data-driven approach to making decisions in a variety of settings using a fusion of data from North America and Europe. In each of the aforementioned studies, the authors have developed models relying on uncertain estimates of parameters that govern the disease dynamics such as infection rate and mortality rate. Our approach complements these decision-making models by providing an alternative estimate of geographic burden, which, importantly, is not conditional on uncertain disease dynamics and instead is based on publicly-available data that can be regularly obtained regardless of global pandemic status. We refer the interested reader to Thul and Powell [66] and Yaesoubi and Cohen [75] for example works that study optimal resource allocation under uncertainty using an SIR framework based on uncertain estimates of parameters which govern the disease dynamics.

Outside of decision making and optimization, Beard et al. [6] reviewed spatial decision support systems (SDSSs) among zoonotic disease outbreaks for regional risk prediction. Devarakonda et al. [17] leveraged concepts from multiple criteria decision analysis to produce regional risk estimates of vector-borne infectious diseases. Our approach is sufficiently general to be applied in settings similar to these, but has the benefit of overcoming pitfalls associated with time-based epidemic data.

Lastly, the work most closely aligned with ours is that of Stolerman et al. [65]. In Stolerman et al. [65], the authors defined COVID-19 activity at the county level as a function of the epidemiological reproduction number. Using only internet-based digital traces as input data, the authors measured the performance of their ML approach to anticipate sharp increases in COVID-19 activity. However, Stolerman et al. [65] performed their analysis on a total of 97 US counties whereas our approach is designed to identify high-risk counties from a much larger pool of 3140 US counties. Further, our approach leverages county-level characteristics that the US Census Bureau makes regularly available and accessible. Finally, our approach differs from that of Stolerman et al. [65], which was designed to predict sharp increases in COVID-19 activity, because we evaluate the potential of ML algorithms to predict the impact of emerging variants of SARS-CoV-2 using outcomes from existing variants.

## Data curation

In this section, we describe how we extract the data for our models’ dependent variables and features. We further describe the definition of dependent variables (i.e., county-level outcomes) analyzed by our ML approach.

### Data extraction

We used two categories of county-level data to train and validate our pandemic burden classification models. The first category, SARS-CoV-2 Variant Outcomes, comprises the dependent variables of our study. The second category, US County Data, contains the features we considered for final model selection.

#### Extracting SARS-CoV-2 variant outcomes data (Dependent Variables)

For 3140 US counties, we extracted outcomes from the COVID-19 Data Repository by the Center for Systems Science and Engineering (CSSE) at Johns Hopkins University (JHU) [see 18] from April 2020, for the Alpha variant of SARS CoV-2 virus, and from November 2021 to March 2022 for the Omicron variant. We further define how we processed these data to create our study’s dependent variables for the Alpha and Omicron SARS-CoV-2 variants in Sections 3.2.1 and 3.2.2, respectively.

In summary, we collected county-level outcomes for two unique periods of the US COVID-19 pandemic where the emergence of a variant of the SARS-CoV-2 virus occurred.

#### Extracting US county data (Features)

We included county-level economic and demographic (e.g., gender, age, race, household, education, income, and work commute) population estimates from the American Community Survey (ACS) from 2014-2018 [11] provided by the US Census Bureau and health data provided by the County Health Rankings & Roadmaps program [60] as candidate features for our ML algorithms. We calculated the Haversine distance from a county’s census-reported latitude and longitude to all 488 US airports [33] with scheduled airline service and assign the minimum distance, in kilometers (km.), for each county as an additional feature. We considered the average temperature (°F) and total precipitation (in.) for each county in each month of 2019 available from a research group’s public repository [39]. We also considered the factors associated with COVID-19 incidence from the third table of a recent study [37] but excluded two factors: 1) the primary care clinicians other than physicians per 10k population, which is not significantly associated with COVID-19 incidence, and 2) the Gini Income Inequality Index whose number of county-level estimates as of 2018 was less than 1000 across the US.

Overall, we curated a comprehensive list of all baseline and temporal feature names, descriptions, and data sources included in our study in eTable 1 of the Supplemental Material.

### County-level outcome definitions

To determine whether the incidence of future variants of a novel virus were predictable, we defined a county-level dependent variable using two unique, dominant variants of SARS-CoV-2 in the US; one for use first in training and then in validating our ML models (i.e., Alpha variant) and one for use to validate their performance (i.e., Omicron variant).

#### Alpha variant outcome definition

We defined the 100 days since January 20, 2020 (i.e., the first confirmed US case of COVID-19 [31]) as the study period for training our ML algorithms, and defined a binary training dependent variable. Specifically, we denoted the counties that belong to the upper quartile of cumulative, confirmed COVID-19 cases per 100k population at the end of the study period defined above as positive and all other counties as negative. We specifically chose the 100-day period for two reasons: 1) on the 100th day, there existed sufficient stability in early SARS-CoV-2 testing data; 2) a recent pandemic preparedness proposal [41] from the White House, called, “American Pandemic Preparedness: Transforming Our Capabilities” calls for the design, testing, and authorization of vaccines “within 100 days after the recognition of a potential emerging pandemic threat.”

We chose the upper quartile of the distribution of viral incidence to separate counties that experienced high pandemic burden from those that did not. As such, those counties that belong to the upper quartile represent the top 25% of viral incidence over the 100 days following the Alpha variant’s introduction in the US. Alternative distributional splits could be considered and may include selecting the top 1%, 10%, or top 50%, separated using the median. In the end, we selected the top 25% as a trade-off between imbalanced category distributions for our supervised learning problem, where predicting the top 1% represents a highly-imbalanced and challenging prediction problem with the utility of separating groups to guide the allocation of scarce medical resources, where predicting the top 50% may not be specific enough and lead to ambiguity over the most at-need counties. We propose that alternative outcome variable definitions could be considered as important future research directions described in Section 6.

#### Omicron variant outcome definition

To conduct an appropriate prospective validation of our data-driven prediction models, we utilized an equivalent dependent variable for the emergence of the Omicron variant to the dependent variable definition for the Alpha variant. In particular, we defined the 100 days since November 22, 2021 (i.e., the first confirmed US case of the Omicron variant [51]) as the study period for prospectively validating our ML algorithms. For this study period, we defined a binary dependent variable that denoted the counties that belong to the upper quartile of the change in cumulative, confirmed COVID-19 cases per 100k population from the end of the study period (i.e., March 2, 2022) defined above to the beginning of the study period (i.e., November 22, 2021) as positive and all other counties as negative.

## Methods

In this section, we describe the ML algorithm choices we made, and offer an overview of our model training procedure, including the classification and ranking ML algorithms we implemented for use in predicting future outcomes for the emerging SARS-CoV-2 Omicron variant.

### Algorithm choice and performance metrics

We chose to implement and compare a ML classifier, specifically Stepwise Forward Selection Logistic Regression (SFLR), and a ranking learner, specifically SGBAP [21]. The model training approach we propose below is flexible insofar as there are many choices for ML classifiers and ranking learners for use in training models. We chose SFLR as our main classifier for its ability to measure the feature-level contribution to model performance and its standard applicability in statistical packages. A reasonable alternative to SFLR is Stepwise Backward Selection Logistic Regression (SBLR), which meets the same criteria as SFLR for being included in our experimental results. Random Forest (RF) models can also report feature importances and are widely available in statistical packages. We conducted an additional set of experiments that included SFLR, SBLR, and RF classifiers and reported those results in eTable 3 in the Supplemental Materials. For the sake of simplicity and conciseness, we will use SFLR as the main classifier for the remainder of the paper and describe its implementation in further detail in Section 4.3.

While classifier and ranking learner algorithms can easily be applied in a supervised learning setting, they differ in the objective we train them to optimize. We explain these objective function differences below and then describe how we benchmark their predictive performance.

Classifiers are algorithms that are trained to learn patterns from data which optimally characterize instances into unique classes [36]. Once trained and validated, classifiers with good performance can be used by policymakers to assign instances to unique classes; in this paper, we propose that a classifier could be used by a policymaker to determine whether a county will belong to the class of counties that will experience the upper quartile of pandemic burden at the onset of an emerging variant of SARS-CoV-2. We chose to benchmark the performance of a classifier on this task using the area under the receiver-operating characteristic curve (AUROC) metric, which has been widely used to evaluate the performance of classifiers [29].

Ranking learners are a natural fit to problems where the goal is to prioritize instances. In this county-level viral burden prediction problem, we aim to simulate the utility of ranking learners in prioritizing the counties predicted to experience the largest viral incidence. Ranking learners are trained to optimize a metric that measures ranking performance; most often, this metric is a pairwise loss function which aims to rank every instance whose outcome is positive-labeled above every instance whose outcome is negatively-labeled in a supervised learning setting with binary outcomes. Since our classification algorithm implicitly optimizes this pairwise loss, we instead implement a ranking learning that optimizes the average precision (AP) metric. AP is a listwise ranking metric which, when optimized, prioritizes the order of the highest-ranked instances over the pairwise ranking of positive and negative instances [12]. We hypothesized that a trained and validated ranking learner with an AP loss function will assist a policymaker in identifying the top *k* highest-ranked counties likely to experience pandemic burden at the onset of an emerging variant of SARS-CoV-2. By identifying the top *k* highest-ranked counties with good performance, the learner could serve a policymaker by suggesting which counties should receive scarce medical resources to combat increasing viral spread.

We benchmark the performance of a ranking learner on this task using two metrics and they are as follows. The first benchmark we propose using to evaluate a ranking learner’s performance is the Precision@*k* metric, which has been previously used to evaluate the performance of ranking learners [21]. Briefly, Precision@*k* measures the ratio of positively-labeled counties among the largest *k* predicted function values that our models return; thus, Precision@*k* summarizes the percentage of at-need counties that the top *k* function values our models return correctly identify. The second benchmark we propose for evaluating a ranking learner’s performance is the Recall@*k* metric. Previous literature [10] identified that an inherent trade-off exists between a learner’s precision and recall performance, so we choose to evaluate a learner’s on both precision and recall to establish its performance. Recall@*k* measures the ratio of positively-labeled counties among the *k* counties with the largest population-adjusted viral incidence; thus, Recall@*k* summarizes the percentage of the top *k* at-need counties that a learner correctly identified. Now that we have established the motivation for the use of our ML algorithms, we introduce the procedure we used to train them.

### Model training procedure

**Fig. 1:**
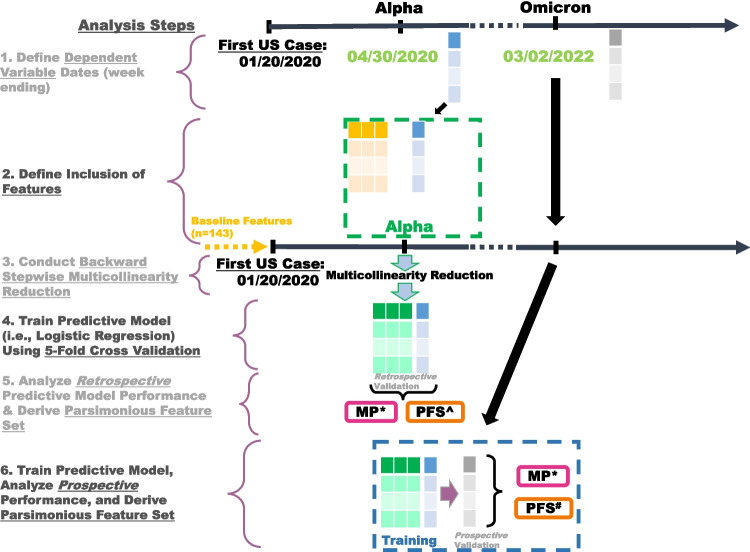
The analytic flow of defining features and dependent variables for the study and training and evaluating our predictive models. See all of the model performances presented here in Tables 2 and 3. *MP = Model Performance. $^\wedge$PFS = Parsimonious Feature Set. $^\#$See results of *Prospective* Parsimonious Feature Set procedure, trained on Alpha outcomes in Table 4

After collecting baseline and variant-specific temporal data to form a set of candidate features for use in training our ML models, we followed the backward stepwise variance inflation factor (VIF) procedure [16], described below in Section 4.2.1 to address their potential multicollinearity. We then used ML algorithms, specifically Random Forest, SFLR, and SGBAP, to train our models and validate their performance on hold-out testing data. We retrospectively validate (i.e., conduct an internal validation) according to a stratified, 5-fold cross validation procedure, described below in Section 4.2.2, by measuring the AUROC, Precision@*k*, and Recall@*k* metrics. We use a cross-validation approach to establish that our approach is capable of learning patterns from its training data and applying it out-of-sample. Further, a cross-validation approach allows us to generate confidence intervals for model performance metrics. In addition to a retrospective validation, we conduct a prospective validation (i.e., an external validation) of our models. We begin the prospective validation by training models using all 3,140 US counties and Alpha variant outcomes. We apply these trained models’ preidctions to the Omicron variant outcomes for all 3140 US counties and measure their classification and ranking performance using the AUROC, Precision@*k*, and Recall@*k* metrics. We provide in Fig. 1 a depiction of the steps of the analysis we conducted for this study.

In summary, we trained our ML algorithms using the binary dependent variable defined in Section 3.2.1 and validated their performance using hold-out county-level outcomes of two unique, dominant variants of SARS-CoV-2 in the US previously defined in Sections 3.2.1 and 3.2.2. We summarized the feature differences among the upper quartile counties for the Alpha and Omicron with a random subset of counties using *t*-tests. We conducted our analysis using Python 3.8.3 [28] and applied our ML algorithms and measured their AUROC performance with the scikit-learn [56] Python package. The 95% confidence intervals (CIs) of mean model performance across five stratified folds we report are calculated using the standard error of the mean and the appropriate multiplicative factor according to a Student’s t-distribution.

#### Backward stepwise multicollinearity reduction

Given the large number of features we collect for each county, we use a backward stepwise selection method [16] to assess multicollinearity using each feature’s variance inflation factor (VIF). The VIF of a feature is a function of that feature’s $R^2$ regression score against the remaining features in the feature set [36]. As a result, the magnitude of the largest VIF value amongst a feature set indicates the presence of multicollinearity.

We pre-process our data by reducing the main set of features collected as described in Section 3.1.2 and listed in eTable 1 of the Supplemental Material using a backward stepwise selection method that removes features one-at-a-time until the maximum VIF value in the remaining feature set is less than a user-specified value. Larger VIF values indicate a strong presence of multicollinearity. Thus, smaller VIF value cutoffs lead to less multicollinear features which, in turn, lead to stronger assurances that the features we identify as predictive cannot be explained by other features. While James et al. [36] suggest a maximum VIF value of 5 or 10 as acceptable, we select a maximum VIF value of 2. We chose to make a stricter cutoff than what is traditionally recommended given the overlapping nature of the socioeconomic data we collect on US counties. As a result, our decision to choose a lower acceptable maximum VIF value enables us to strictly enforce a limit of multicollinearity in the feature set of the analysis. The VIF metric is computed agnostic of the ML algorithm we choose for the selected feature. As a result, we can conduct our backward stepwise selection procedure before we train any ML models and instead use the selected feature set resulting from the procedure to train our models.

#### Stratified 5-fold cross validation procedure

When training our models, we split 3140 US counties into five groups each stratified by equal proportions of the dependent variable used in training our models. We train each model with four of five groups and evaluate the performance of the trained model on the fifth group, and then repeat this procedure five times, each time choosing a unique hold-out testing group, so that each of the five unique stratified groups will belong to the hold-out evaluation set once. In this approach, the average hold-out model performance we report is representative of all counties in the US, see eFigure 1 in Supplemental Material.

### Classification algorithm: Stepwise forward selection logistic regression

We chose a stepwise forward selection classification algorithm (i.e., SFLR) for two reasons. The first reason we chose a forward stepwise approach was that it enables us to measure the association of the addition of features to the model to the classification criteria; as each feature is added to the model in a forward stepwise manner, we can calculate the performance benefit it contributes and deliver a parsimonious feature set from the resulting model. The second reason we chose a SFLR approach is because there exists a variety of standard statistical software (e.g., SAS [64] and R [78]) capable of its implementation. By selecting an algorithm that could be implemented in a variety of analysis workflows, we have aimed to make our approach as accessible as possible to analysts that support policy making and decision making. Finally, we will note that recent work by Hastie et al. [30] has highlighted that forward selection methods still perform comparably to more advanced and less accessible best subset selection methods.

We now describe in detail the process by which features are added stepwise to a logistic regression model.

#### Forward stepwise feature addition - Feature importance

We seek to attribute the importance of the county-level features we collected to a LR classifier’s AUROC scores. We propose that features selected based on their contribution to AUROC performance of LR classifiers on its training data may make up a parsimonious feature set for further consideration by decision makers. Our approach differs slightly from standard forward stepwise approaches [36], which use metrics like p-values or Akaike Information Criterion (AIC) as a basis for including a feature into the final model, by instead using the AUROC metric to decide which features will be included in the final model. This aligns with our modeling goal of accurately classifying counties by their pandemic burden.

The feature addition process for our models proceeds as follows. We train and validate individual LR classifiers for each member of the statistically selected subset of features described in Section 4.2.1, select the feature that achieves the largest AUROC score in the training data, and remove it from the pool of feature candidates for model addition. Next, we train individual LR classifiers using the previously selected feature and each of the remaining members of the statistically selected subset of feature. After we select the remaining feature that achieves the largest AUROC score on the training data, we remove the newly selected feature from the pool of feature candidates for addition to the model since it was previously selected for addition. We continue this sequential process until we find that adding features no longer improves the AUROC score of the LR classifier on the training data. The stepwise feature addition process is replicated for each of five unique validation folds, as described in Section 4.2.2.

### Ranking algorithm: Stochastic gradient boosted average precision

We implement SGBAP with an exponential-based surrogate of an AP loss function designed by Frery et al. [21]. In addition to the utility of this algorithm that we outlined in Section 4.1, SGBAP optimizes the loss function $1-\hat{AP}_{exp}$ in the function space *f*(*x*) as opposed to the parameter space because optimizing in the function space allows for the use of custom loss functions for optimization.

Into SGBAP, we input *M* training data instances $Z=\{(x_i,y_i)\}_{i=1}^M$ where $x_i \in X$ is the instance’s unique feature vector and $y_i \in \{0,1\}$ is the instance’s class label. $y_i=1$ are positively-labeled instances, and $x_i^+$ its respective feature vector, and we let *P* be the number of these examples; conversely, $y_i=0$ are negatively-labeled instances, and $x_i^-$ its respective feature vector, and we let *N* be the number of these instances such that $P+N = M$.

Briefly, as Frery et al. [21] described, gradient boosting is a method that sequentially and adaptively optimizes a loss function using a linear combination of weak learners. At step *t*, the function value of SGBAP $f_t(x)$ is updated as follows.

1 $$\begin{aligned} f_t(x) = f_{t-1}(x)+\gamma _t h_t(x) \end{aligned}$$

where $h_t \in \mathcal {H}$ is a model belonging to a set of regression trees $\mathcal {H}$, which were chosen according to the seminal introduction of stochastic gradient boosting by Friedman [22] and $\gamma _t$ is a line search parameter that weights the performance of $h_t$ in updating the loss function $\ell (Z,f_{t-1}(x))$. The loss function $\ell (Z,f_t(x))$ calculates the loss in average precision of *f*(*x*) that results from the training data *Z* input at step *t*. In our implementation of SGBAP, we follow a mini-batch procedure during training. Mini-batch procedures are recommended for use in gradient boosting methods to improve computational performance; specifically, we randomly sample a ratio $\lambda \in [0,1]$ of training examples and denote the mini-batch, of size $\lambda M \in \mathbb {Z}:\lambda M \in [1,M]$, $Z^\prime = \{x_i^\prime ,y_i^\prime \}_{i=1}^{\lambda M}$ at each of our algorithm’s *T* iterations. We implement the gradients for the loss function called $1-\hat{AP}_{exp}$ for data with positive and negative labels derived by Frery et al. [21] $g(x^+)$ and $g(x^-)$, respectively. The full SGBAP algorithmic procedure we implement is presented in Algorithm 1 below. We train and subsequently validate this algorithm on each of five unique validation folds, as described in Section 4.2.2.

**Algorithm 1:**
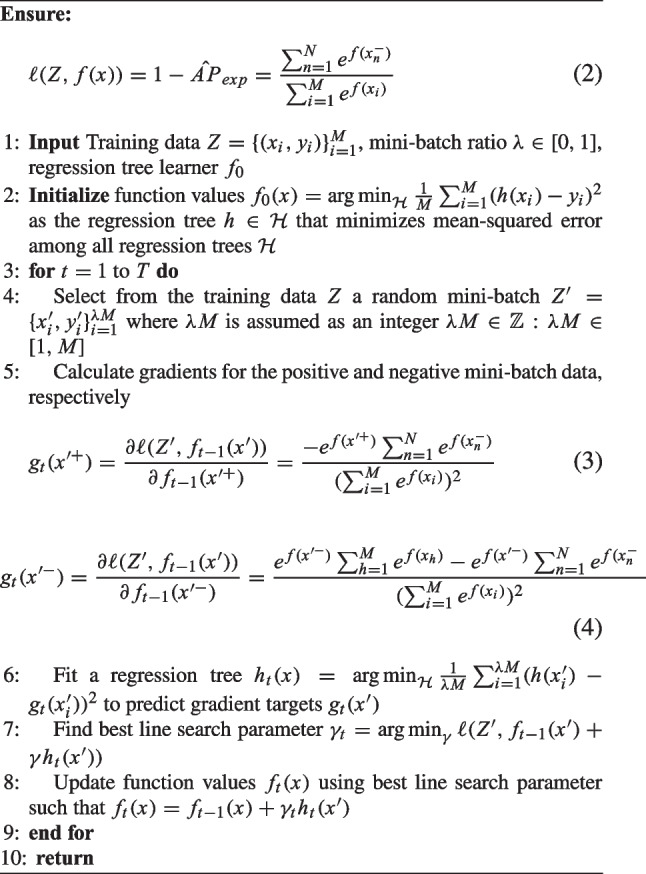
SGBAP with Exponential-based Surrogate of AP.

## Results

In the subsequent sections, we provide results on Alpha and Omicron variant outcomes at the county level, introduced previously in Sections 3.2.1 and 3.2.2, a multicollinearity-driven reduction of feature subsets, ML classifier and ranking learner performance, and a feature importance analysis. Further, we compare these models to four benchmarks: 1) “Random Guess”: a random selection of 25% of US counties as a prediction of the upper quartile, 2) “Population Density”: a selection of the top 25% of most population dense US counties as a prediction of the upper quartile, 3) “Alpha Cases”: a selection of the top 25% US counties with the most cases per 100k population of the previous (i.e., Alpha) variant, and 4) “Random Forest”: a powerful data-driven model that chose to include because of its utility in other studies with respect to variable inclusion, high discrimination-scored results comparable to more complicated methods, and computational efficiency [9]. Random Forest models have two notable hyper parameters: number of trees and the proportion of features to be selected from at each tree’s node split. We chose to train our Random Forest models with 1500 trees and $\sqrt{p}$ proportion of features to be selected from at each tree’s node split. In eTable 4 of the Supplemental Material, we conducted a Hyperparameter Optimization routine, developed using Bayesian optimization, to test the hypothesis that optimized hyperparameters lead to improved model performance. In the case of our work, optimizing the hyperparameters of the LR and RF models led to modest performance improvements. As a result, our final models’ hyperparameters are not optimized and standard values are used instead. In eTable 5 of the Supplemental Material, we conducted a sensitivity analysis by applying two standard approaches to balancing imbalanced datasets for classification, namely: 1) oversampling the minority class (Oversampling) and 2) applying a class weight to match the class distribution of the data (Class Weight) to evaluate whether these techniques could improve the discrimination performance of our models. In this work, we did not observe statistically significant differences when applying these imbalance correction techniques during the training of our models and, given the imbalanced dataset approach routine increases runtime considerately and complicates the model pipeline for an analyst, we chose not to include imbalance correction techniques in the main study.

### Results of multicollinearity reduction analysis

After collecting 143 baseline features, we reduced the size of the set of features to 41 by enforcing a strict maximum VIF value of two. The selected features and respective final VIF values after conducting the backward stepwise selection method are listed in eTable 2 of Supplemental Material and in Table 1 where the features are compared across county-level outcomes of the Alpha and Omicron variants.

### Results of county-level outcomes for each SARS-CoV-2 variant

Below, we include statistics that summarize the county-level outcomes for two unique SARS-CoV-2 variants in the US.

#### Alpha variant outcomes at the county level

Among the 3140 US counties we analyzed in this study, the average number of population-adjusted confirmed cumulative COVID-19 cases was 153 while the median was 59 at end-of-day on April 30, 2020, 100 days since the introduction of SARS-CoV-2 to the US. Counties with greater than 151 confirmed cumulative COVID-19 cases, adjusted for population, made up the upper quartile of cumulative, confirmed COVID-19 cases per 100k population on April 30, 2020.

#### Omicron variant outcomes at the county level

We observed significant deviation among the change in cumulative, confirmed COVID-19 cases per 100k population from November 22, 2021 to March 2, 2022, the 100-day period after the national introduction of the Omicron variant to the US. Among our study’s 3,140 counties, 756 reported greater than 10,000 cases per 100k population; in other words, about 24% of US counties reported that over 10% of their populations had confirmed COVID-19 infection between November 22, 2021 and March 2, 2022. All of those counties and 29 others (i.e., 785 total) make up the upper quartile of the change in cumulative, confirmed COVID-19 cases per 100k population between November 22, 2021 and March 2, 2022.

#### A feature comparison for counties in the upper quartile of alpha and omicron variant incidence

In Table 4, we compare the means of 41 features - that make up the final feature set that resulted from the multicollinearity reduction analysis - associated with the counties that belonged to the upper quartile of Alpha outcomes as described in Section 5.2.1, those of the upper quartile for Omicron outcomes, and a random selection of 785 counties from the 3140 counties we studied.

**Table 1:** A comparison of the 785 counties that each make up the upper quartile for the Alpha and Omicron variants with a random selection of 785 of our study’s 3140 counties

Feature	Alpha: UQ$^\textrm{a}$	Omicron: UQ$^\textrm{a}$	Random 785 Counties	p
Number of Counties	785	785	785	–
Population Density (Per Sq. Mile) (mean (SD))	661.84 (3484.10)	486.97 (3351.49)	221.16 (737.45)	0.008
Area (Land) (mean (SD))	781.49 (1186.77)	1434.87 (6637.34)	1133.37 (5364.66)	0.034
% Total Population: 15 to 17 Years (mean (SD))	3.92 (0.58)	3.92 (0.61)	3.91 (0.61)	0.986
% Total Population: 35 to 44 Years (mean (SD))	11.96 (1.45)	11.96 (1.31)	11.56 (1.51)	<0.001
% Total Population: 45 to 54 Years (mean (SD))	13.22 (1.39)	13.12 (1.57)	12.95 (1.53)	0.001
% Total Population: Native Hawaiian and Other Pacific Islander Alone (mean (SD))	0.06 (0.13)	0.11 (0.64)	0.09 (0.39)	0.035
% Total Population: Some Other Race Alone (mean (SD))	2.35 (3.88)	2.11 (3.90)	2.04 (4.00)	0.275
% Total Population: Two or More Races (mean (SD))	2.23 (1.40)	2.50 (2.26)	2.42 (1.93)	0.015
% Family Households: Male Householder, No Wife Present (mean (SD))	4.79 (1.34)	4.91 (1.65)	4.58 (1.46)	<0.001
% Renter-Occupied Housing Units: 2-Person Household (mean (SD))	26.34 (4.43)	26.20 (4.50)	26.43 (5.19)	0.644
% Renter-Occupied Housing Units: 3-Person Household (mean (SD))	15.40 (3.80)	15.47 (4.03)	14.52 (4.45)	<0.001
% Renter-Occupied Housing Units: 4-Person Household (mean (SD))	11.23 (3.56)	11.31 (3.74)	11.01 (3.83)	0.263
% Renter-Occupied Housing Units: 5-Person Household (mean (SD))	5.67 (2.64)	5.60 (2.80)	5.61 (3.02)	0.87
% Renter-Occupied Housing Units: 6-Person Household (mean (SD))	2.36 (1.74)	2.27 (1.71)	2.30 (1.90)	0.559
% Renter-Occupied Housing Units: 7-or-More Person Household (mean (SD))	1.37 (1.34)	1.39 (1.68)	1.41 (1.77)	0.883
% Population 25 Years and Over: Some College (mean (SD))	29.05 (4.69)	29.88 (5.17)	30.74 (5.21)	<0.001
% Population 25 Years and Over: Doctorate Degree (mean (SD))	0.98 (0.94)	0.84 (0.80)	0.84 (0.90)	0.002
% Workers 16 Years and Over: Carpooled (mean (SD))	9.47 (2.78)	9.43 (2.65)	9.67 (2.71)	0.176
% Workers 16 Years and Over: Motorcycle (mean (SD))	0.11 (0.16)	0.12 (0.16)	0.13 (0.19)	0.021
% Workers 16 Years and Over: Bicycle (mean (SD))	0.34 (0.73)	0.33 (0.67)	0.35 (0.73)	0.87
% Workers 16 Years and Over: Other Means (mean (SD))	1.01 (0.85)	1.18 (2.42)	1.07 (1.31)	0.135
% Workers 16 Years and Over: Did Not Work At Home: 20 to 29 Minutes (mean (SD))	17.15 (5.06)	16.36 (5.28)	15.64 (5.56)	<0.001
% Workers 16 Years and Over: Did Not Work At Home: 30 to 39 Minutes (mean (SD))	14.05 (4.74)	13.25 (5.16)	12.74 (5.10)	<0.001
% Workers 16 Years and Over: Did Not Work At Home: 60 to 89 Minutes (mean (SD))	5.69 (3.83)	5.00 (3.52)	4.73 (3.17)	<0.001
% Workers 16 Years and Over: Did Not Work At Home: 90 or More Minutes (mean (SD))	2.78 (2.01)	2.55 (1.91)	2.50 (1.77)	0.007
Percent of Persons Under 19 Years Without Insurance (2013 est.) (mean (SD))	5.25 (2.83)	5.07 (2.93)	6.31 (3.78)	<0.001
Percent Diabetics (Adults) (mean (SD))	12.44 (4.37)	12.38 (4.26)	12.09 (4.15)	0.23
Percent of Persons with Limited Access to Healthy Foods (mean (SD))	7.54 (6.67)	7.88 (7.21)	8.90 (8.85)	0.001
Percent of Persons with Access to Exercise Opportunities (mean (SD))	65.54 (24.84)	65.07 (23.25)	63.20 (23.19)	0.12
Percent Obese Persons (20 Years and Over) (mean (SD))	32.98 (6.23)	33.16 (5.85)	32.69 (5.47)	0.281
Minimum distance (in km) to an airport with scheduled service (mean (SD))	24.29 (21.60)	30.06 (48.72)	33.62 (46.49)	<0.001
Mar Precipitation / inch (mean (SD))	2.99 (1.20)	3.08 (1.46)	2.76 (1.46)	<0.001
Apr Precipitation / inch (mean (SD))	4.77 (2.40)	4.46 (2.23)	4.16 (2.26)	<0.001
May Precipitation / inch (mean (SD))	5.09 (2.76)	5.51 (3.24)	5.44 (3.20)	0.015
Jul Precipitation / inch (mean (SD))	4.36 (2.24)	4.16 (2.11)	3.80 (2.20)	<0.001
Aug Precipitation / inch (mean (SD))	3.97 (2.03)	4.08 (2.29)	3.83 (2.30)	0.077
Sep Precipitation / inch (mean (SD))	1.93 (2.08)	2.27 (2.65)	2.57 (2.53)	<0.001
Nov Precipitation / inch (mean (SD))	2.45 (1.20)	2.86 (2.08)	2.53 (2.15)	<0.001
Unemployment rate (mean (SD))	6.23 (2.81)	6.34 (3.04)	5.69 (2.87)	<0.001
People in institutionalized group residences (mean (SD))	3.54 (4.45)	3.65 (4.92)	3.53 (4.69)	0.853
Intensive care unit beds, No. per 10 000 population (mean (SD))	44.90 (134.33)	32.85 (111.93)	27.37 (113.95)	0.013

We present a total of 41 features, which are the result of conducting a multicollinearity reduction analysis on 143 baseline features and are considered for final SFLR model selection. Only 12 of the 41 features we present here are not statistically significant at the $p = 0.05$ level, thus providing strong evidence that classifying or ranking one of these subsets well does not guarantee good classification or ranking performance on another

$^\textrm{a}$UQ = upper quartile; see Sections 5.2.1, 5.2.2

Compared to the set of counties in the upper quartile of Alpha outcomes, the counties in the upper quartile of Omicron outcomes had significantly larger land area, Native Hawaiian and Pacific Islander populations, workers with “Other Means” of transit to work (i.e., workers that did not use a car, truck, van, public transit service, motorcycle, bicycle, or walking path to transit to work), kilometers to travel to the nearest airport with scheduled service, and 2019 precipitation (in in.) for the months of March, September, and November. In contrast, upper quartile Omicron counties had lower population densities, percentages of population with doctoral degrees, percentages of the population with commutes of 20 to 29 minutes, 30 to 39 minutes, 60 to 89 minutes, 90+ minutes, percentages of persons under 19 years of age uninsured, and number of ICU beds.

Both sets of upper quartile counties differed significantly from a random selection of 785 of our study’s counties with larger percentages of the population aged 35 to 44, 45 to 54, percentages of households owned by unmarried men, percentages of 3-person households, and number of ICU beds. Additionally, the upper quartile counties had lower percentages of persons under 19 years of age uninsured and with limited access to healthy foods when compared with the random set of 785 US counties.

### Machine learning model performance results

We followed the analysis procedure outlined in Section 4.2 for each of two dominant variants of SARS-CoV-2 in the US. A summary of our models’ prospective validation performance is presented in Table 3 where the values in bold belong to the models with the best validation performance. The retrospective validation performance results of Table 2 are averaged across a stratified, five-fold cross validation design. The validation results of Table 2 are calculated on out-of-sample data or data that was unseen by the algorithms during model training.

**Table 2:** A summary of classification and ranking metric out-of-sample performances by model on Alpha variant data

		Out-of-Sample Alpha Performance			
Model	AUROC	P@50	P@100	R@50	R@100
Random Guess	0.500	0.250	0.250	0.250	0.250
Population Density	0.656 (0.595-0.717)	0.524 (0.340-0.708)	0.452 (0.354-0.550)	0.416 (0.236-0.596)	0.392 (0.304-0.480)
Random Forest	**0.776 (0.732-0.821)**	0.780 (0.524-1.000)	0.636 (0.533-0.739)	**0.676 (0.580-0.772)**	**0.594 (0.514-0.674)**
SGBAP	0.680 (0.627-0.734)	0.624 (0.375-0.873)	0.526 (0.361-0.691)	0.524 (0.344-0.704)	0.462 (0.345-0.579)
SFLR$^\textrm{a}$	0.697 (0.585-0.809)	**0.796 (0.552-1.000)**	**0.730 (0.555-0.905)**	0.552 (0.361-0.743)	0.488 (0.292-0.684)

We present 95% confidence intervals in parentheses following the mean value obtained from five unique, stratified cross-validation splits. While Random Forest, SFLR, and SGBAP are data-driven algorithms, Population Density is a single feature and we evaluate it herein to serve heuristically (i.e., if a decision maker had only this data available at the time of their decision) as a comparison. For all seven metrics we evaluate, data-driven models (i.e., Random Forest and SFLR) achieve better performance when compared with heuristics (i.e., Random Guess and Population Density). As a result, data-driven models could serve as a critical tool for policymaker intervention decision support at the onset of emerging viral variants

$^\textrm{a}$SFLR = Stepwise Forward Logistic Regression; see Section 4.3

**Table 3:** For these results, we trained each model using all 3,140 counties and their Alpha outcomes

		Out-of-Sample Omicron Performance					
Model	AUROC	P@50	P@100	P@500$^\textrm{a}$	R@50	R@100	R@500$^\textrm{a}$
Random Guess	0.500	0.250	0.250	0.250	0.250	0.250	0.250
Population Density	0.588	0.380	0.360	0.314	0.120	0.200	0.268
Alpha Cases$^\textrm{d}$	0.555	0.480	0.410	**0.342**	0.200	0.260	0.274
Random Forest	0.583	**0.580**	**0.480**	0.318	0.200	0.260	0.274
SGBAP	0.595	0.440	0.370	0.304	**0.320**	**0.360**	0.282
SFLR$^\textrm{b}$	**0.608**	0.380	0.390	0.328	0.300	0.330	**0.302**$^\textrm{c}$

We applied each model’s predictions to the Omicron outcomes for all 3,140 counties and calculated their classification and ranking metric performances. While SFLR and SGBAP are data-driven algorithms, Population Density and Alpha Cases are single features and have been evaluated herein to serve heuristically (i.e., if a decision maker had only this data available at the time of their decision) as a comparison. For six of seven metrics we evaluate, data-driven models (i.e., Random Forest, SGBAP, and SFLR) achieve better performance when compared with heuristics (i.e., Random Guess, Population Density, and Alpha Cases). Prospectively, data-driven models could lead to improved outcomes that result from policymaker interventions at the onset of emerging viral variants

$^\textrm{a}$We chose to calculate these metrics up to 500 counties for the Omicron validation data *only* (i.e., not for the Alpha data) because it best demonstrates how a policymaker may prospectively use our models trained on previous variants at the onset of an emerging variant

$^\textrm{b}$SFLR = Stepwise Forward Logistic Regression; see Section 4.3

**Fig. 2:**
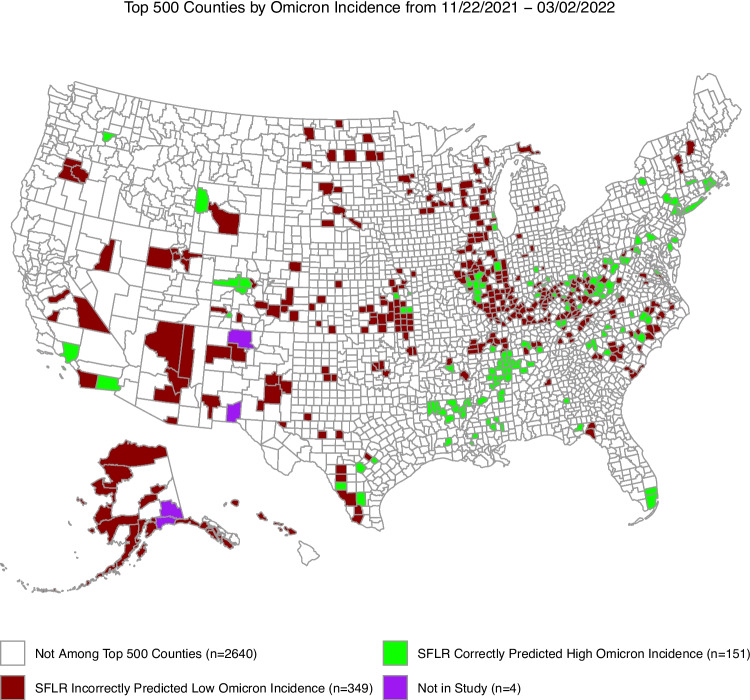
A geographic depiction of the utility of our models. There are 500 US counties not white in color and they are the counties that would experience the largest change in SARS-CoV-2 incidence, after the emergence of the Omicron variant, from November 22, 2021 to March 2, 2022. Among the top 500 counties, those green in color (n=151) were counties our model correctly predicted, in advance, would experience high pandemic burden, defined as belonging to the upper quartile of our change in confirmed cumulative COVID-19 cases at 100 days since the onset of an emerging Omicron variant of SARS-CoV-2. The counties in red (n=349) were counties that our models did not identify as belonging to the upper quartile of Omicron incidence. In this instance, these models are trained on Alpha variant outcomes and validated on Omicron variant outcomes with mean AUROC score 0.598. This plot represents the Recall metric of the top 500 counties by Omicron incidence with a Recall@500 of 0.302. With a few exceptions, clusters of red counties are geographically close to green counties. To use our data-driven model in advance of Omicron’s arrival, one can identify clusters of counties predicted to experience high burden and allocate appropriate resources accordingly

$^\textrm{c}$We generate a geographic depiction of this result in Fig. 2

$^\textrm{d}$We include results for this feature here but not in the Alpha validation data of Table 2 because all values are trivially equal to 1 as a result of the Alpha validation outcome definition. We propose that a policymaker may alternatively use the results of the last variant’s wave as a heuristic to allocate scarce resources

The SFLR cell of the “P@100” column in Table 2, where the score: 0.730 is displayed, represents the mean Precision@100 of models trained and validated on held-out Alpha variant county-level outcomes across five stratified cross-validation folds of US counties. This value indicates that, on average, of the top 100 SFLR model predictions for held-out data, 73% belonged to the upper quartile of Alpha variant incidence. To compare, the SFLR model scored a Precision@100 of 0.390 on out-of-sample Omicron validation data as demonstrated in Table 3. One reason why SFLR performed better on out-of-sample Alpha data when compared with out-of-sample Omicron validation data could be a generalization gap. SFLR is an algorithm that selects features according to their contribution to training data discrmination (i.e., contribution to training AUROC). When applying this SFLR model to out-of-sample Omicron data, the SFLR model does not generalize the patterns it learned well from the training data to the out-of-sample validation data.

The SFLR method exhibited the best performance on the P@50 and P@100 metrics when evaluated using held-out Alpha variant data while the Random Forest method achieved the largest AUROC, R@50, and R@100 values (see Table 2). One surprising result was the larger AUROC score achieved by the Random Forest method compared with SFLR. Since SFLR sequentially adds predictors according to their AUROC until no further contribution can be made, we hypothesized that this method would, on average, score the highest AUROC scores. One reason why the Random Forest method scored higher instead is that the Random Forest method relies on highly nonlinear relationships in the features used for training. As a result, the Random Forest method was able to exploit these highly nonlinear relationships and find patterns in the held-out Alpha variant outcome data that SFLR could not. This result also implies that the relationship between a county’s regularly collected sociodemographic, economic, transportation, and health characteristics (i.e., the features of our data) and the outcomes of the Alpha variant could be better explained by highly nonlinear and complex relationships instead of a log odds-based relationship.

When evaluating our models on held-out Omicron variant validation outcomes, the largest AUROC value was demonstrated by the SFLR method (see Table 3). While the Alpha Cases feature had the largest P@500 metric, Random Forest achieved best performance on the P@50 and P@500 metrics. We calculated that the SGBAP method had the largest R@50 and R@100 metric performances while SFLR scored the highest R@500 values on held-out Omicron validation outcomes. In total, data-driven models like Random Forest, SGBAP, and SFLR had the highest performance metric scores for six of seven metrics on prospective validation. One explanation for why Alpha Cases outperformed data-driven models on the P@500 metric is that many of the counties that experienced high Alpha variant incidence also had higher-than-average Omicron variant incidence (i.e., those counties with high Alpha variant incidence were ranked somewhere between 100 and 500 in Omicron variant incidence). This would result in the Alpha Cases feature achieving the best P@500 score as P@500 is a ranking metric that rewards correct identification of counties that will experience burden.

In general, the data-driven Random Forest, SGBAP, and SFLR models achieve higher validation performance metric scores compared to the Population Density and Alpha Cases heuristics, which are reasonable benchmarks for high-risk county selection in the absence of ML-based models. As expected, both the data-driven models and heuristics significantly outperform the Random Guess across all metrics. Given the challenge of predicting county-level burden caused by the uncertainty of disease dynamics, the result that our data-driven models outperform the Random Guess in every metric we measure may assure the decision maker that making decisions informed by the predictions of our models is better than another decision that may appear based on a random guess.

Figure 2 illustrates how a policymaker might assess the utility of our models for decision-making purposes. In particular, we sorted the change in cases per 100k population from November 22, 2021 to March 2, 2022 at the county level in decreasing order and selected the top 500 US counties. We stratified these 500 counties according to whether our SFLR model correctly predicted that county would belong to the upper quartile of Omicron incidence. Namely, in Fig. 2, the counties in green were counties our model correctly predicted would experience high pandemic burden at the onset of an emerging variant, which we defined as these counties belonging to the upper quartile of the top 500 largest SARS-CoV-2 incidence increase, adjusted for population, from November 22, 2021, to March 3, 2022. The red counties in Fig. 2 were false negative predictions by our model where they belong to the upper quartile of Omicron incidence but were not correctly identified by our model. Despite a few exceptions, our models correctly predicted at least one of the counties among separate clusters of large Omicron incidence; that is, many red counties in Fig. 2 are geographically close to green counties. The close proximity of our model predictions and counties with high Omicron incidence implies that our models are capable of identifying geographic outbreak clusters at the onset of an emerging variant.

**Fig. 3:**
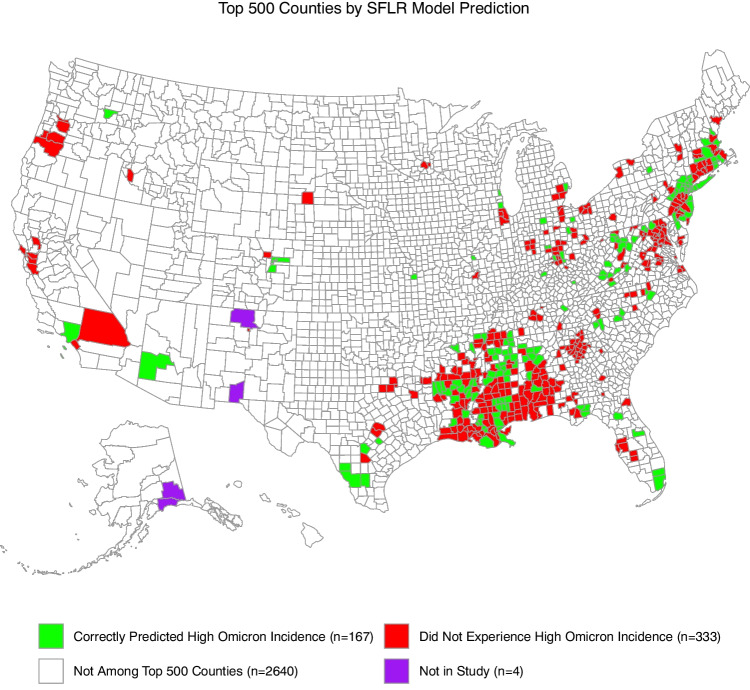
A geographic depiction of the utility of our models. There are 500 US counties not white in color and they are the counties that our SFLR models predicted would experience the largest burden at the emergence of the Omicron variant, from November 22, 2021 to March 2, 2022. Among the top 500 counties, those green in color (n=167) were counties that experienced high pandemic burden, defined as belonging to the upper quartile of our change in confirmed cumulative COVID-19 cases at 100 days since the onset of an emerging Omicron variant of SARS-CoV-2. The counties in red (n=33) were counties that did not belong to the upper quartile of Omicron incidence. In this instance, these models are trained on Alpha variant outcomes and validated on Omicron variant outcomes with mean AUROC score 0.598. This plot represents the Precision metric of the top 500 counties by Omicron incidence with a Recall@500 of 0.328. Red counties could be considered false positive model predictions. We suppose that it would be unlikely that a decision maker sends resources to a large part of the deep south, where a majority of our red counties are located. Thus, this figure illustrates how our model’s prediction could be used in conjunction with a decision maker’s expertise to allocate scarce medical resources in advance of a novel viral variant

Figure 3 provides an alternate way to support decision-making using outputs from our models. It depicts a graph of the Precision@500 metric we measure to analyze model performance. In Fig. 3, we sorted the numeric outputs from our SFLR models at the county level in decreasing order and selected the top 500 US counties. We stratified these 500 counties according to whether they belonged to the upper quartile of Omicron incidence. Namely, in Fig. 3, the counties in green were counties our model correctly predicted would experience high pandemic burden at the onset of an emerging variant, which we defined as these counties belonging to the upper quartile of the SARS-CoV-2 incidence increase, adjusted for population, from November 22, 2021, to March 3, 2022. The red counties in Fig. 3 were false positive predictions by our model where they were predicted to belong to the upper quartile of Omicron incidence but did not experience high Omicron incidence. If used by a decision maker to allocate resources, Fig. 3 highlights that the deep south may have received an excess of resources (i.e., there is a large cluster of red counties in Fig. 3 in the deep South US). Figure 3 could also have been used to send scarce medical resources to the Bay Area of California, but that ultimately this region did not experience high population-adjusted Omicron incidence. Lastly, Fig. 3 demonstrates that our models are capable of providing true positive predictions across the all regions of the US.

### Results of decision-focused analysis

**Fig. 4:**
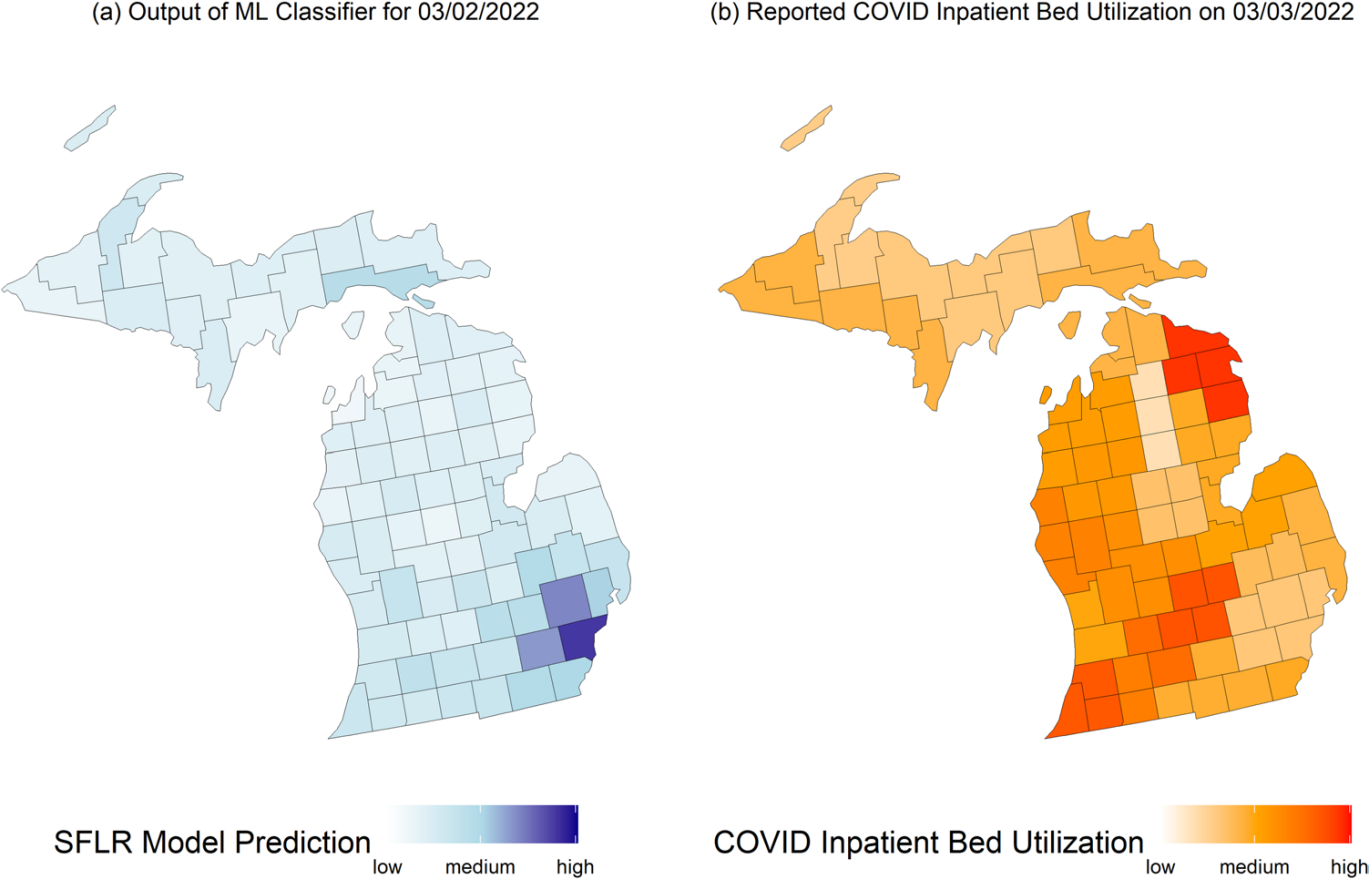
A comparison of the output of our ML classifiers (a) with healthcare utilization (b) on 03/02/2022 – 100 days since the onset of the Omicron variant in the US. A decision maker would have access to the data in subplot (a) on 12/11/2021 and use it to make decisions that could have reduced the burden observed in healthcare utilization rates in subplot (b)

In Fig. 4 below, a decision maker would have access to the data in subplot (a) which are the output of our SFLR classifiers. In particular, a decision maker would likely consider sending scarce medical resources to darker blue regions in subplot (a). In subplot (b), we plotted the inpatient bed utilization among COVID-diagnosed patients by county derived from the CDC COVID-19 Community Level Index data. The data and more information about it is available at https://archive.cdc.gov/www_cdc_gov/coronavirus/2019-ncov/science/science-briefs/indicators-monitoring-community-levels.html. Figure 4(a) may guide decisions around allocating scarce resources whereas Fig. 4(b) demonstrates the outcomes reported after the decisions made using (a). Figure 4 also highlights how policy makers at the state level can leverage our approach to support decisions despite our approach leveraging data from the national level.

### Results of dynamic data sensitivity analysis

In an effort to explore the bounds of our approach’s performance, we experimented with adding to our model time-based features to make them dynamic. To be considered in our sensitivity analysis experiments, the new dynamic data needed to meet the two important criteria below.

1. The new features must have been available at the county-level. In contrast, features that are available only at the state level would limit the predictive value of our models. This is because each county within a state would each have the same value for a feature and cause the model’s predictions to become more homogeneous in theory.
2. The new features must have been available for the Alpha variant. We recognize this is a strict requirement given that the onset of COVID-19 in the US was sudden and that relevant data infrastructure systems were not well-developed by that time. However, we justify this requirement because we propose that our approach could be used for emerging viruses beyond COVID-19. Thus, data sources that would have been available during the Alpha variant of the COVID-19 pandemic are likely to be available at the onset of the next emerging viral disease in the US.

Using these criteria, we identified three new sources of dynamic features to add to our models and complete this sensitivity analysis. The first is the Google COVID-19 Community Mobility Reports data (Google Mobility data), which is a source of data gathered daily across geographic regions and reports a change in baseline of mobility to hotspots for infectious disease like parks, workplaces, and homes. To make use of these data, we gathered 7-day moving averages of Google Mobility data. We collected three moving averages at at least a 3-week lag from the outcome our models were trained to predict to simulate real-world practice where the data may not be immediately available. Specifically, we collected 7-day moving averages of each mobility index for the weeks ending March 21, March 28, and April 4, 2020 for use in training the Alpha variant models whose outcome period ended April 30, 2020. We further collected 7-day moving averages of each mobility index for the weeks ending January 29, February 5, and February 12, 2022 for use in evaluating the Omicron variant models whose outcome period ended March 2, 2022.

The second source of dynamic features is the The Delphi Group at Carnegie Mellon University U.S. COVID-19 Trends and Impact Survey, in partnership with Facebook (CITS data), which gathered survey response data daily and reported COVID-19-like symptoms, behavior, mental health, and economic- and health-related impacts caused by the pandemic. Like the Google mobility data above, we collected this data at at least a 3-week lag from the outcome our models were trained to predict, which resulted in a collection of the April 11, 2020 survey data for the Alpha variant models and February 12, 2022 survey data for the Omicron models. To be specific, we collected survey data that belonged to the Symptoms, Contact, Behavior(s), and CLI/ILI categories for the specified dates.

The third and final source of dynamic features we collected for this sensitivity analysis is the JHU CSSE data. We used this collection of regional COVID-19 incidence data to define our models’ outcomes, and now we propose using lagged data as a feature for the dynamic models we test in this sensitivity analysis. In particular, we collect three 7-day moving average COVID-19 incidence at at least 3-week lag from the outcome our models were trained to predict. Thus, we collected 7-day moving averages of incidence for the weeks ending March 21, March 28, and April 4, 2020 for use in training the Alpha variant models whose outcome period ended April 30, 2020. We further collected 7-day moving averages of each mobility index for the weeks ending January 29, February 5, and February 12, 2022 for use in evaluating the Omicron variant models whose outcome period ended March 2, 2022.

One challenge that is introduced when including new sources of dynamic data to the study is data missingness. To summarize the data missingness of the features we just described, we include a table, for each variant, which lists for each feature the percentage missing from the 3140 counties we originally included in our study.

In the Supplemental Material, eTables 6 and 7 demonstrate significant missingness among the dynamic features we add to our models for this sensitivity analysis. To address this missingness, we propose two methods. In the first method, we propose to conduct a complete-case analysis whereby we remove from the sensitivity analysis any county with at least one missing value in either the Alpha or Omicron data. As it turns out, this method results in zero county that has complete data. This result notwithstanding, even if we were able to conduct the complete-case analysis, we believe we would have introduced a significant bias as the counties which have complete data would not likely be representative of the entire US. This is because the counties where data was collected from all of these dynamic sources would be more likely to have residents with cell phones, where much of the Google Mobility data is sourced, and more likely to respond to surveys, introducing a selection bias.

The second method we propose using to address missingness is imputation. In this method, we propose imputing, or filling with the most likely value, the missing values in the data. However, in the literature, there are well-documented cases where imputation methods have deleterious downstream effects on model results. In particular, models trained using the imputed data exhibit significant bias when compared with the true data in simulation studies. Despite this documented limitation, we proceeded with a sensitivity analysis as follows.

Given our experimental setup, where we split our data into train and test splits, the imputation process is complex to avoid any data leakage problems where information from the test set leaks into the training set via the imputation process. The process to impute the dynamic data proceeded as follows, where we conducted each step for both the Alpha and Omicron variants. First, we collected all the proposed dynamic data and appended it to our original, baseline data which has complete values. Second, we split the data into a 75%/25% train/test split. We used the same split for the Alpha and Omicron data so that we can accurately evaluate our models. Third, we used Random Forest (RF) models to impute missing values in the data using the mice package in R. Fourth, we applied our existing model training procedure on the newly-imputed data. Fifth, we applied our existing model evaluation procedure on the newly-imputed data.

In eTable 4 of the Supplemental Materials, we compared the out-of-sample AUROC performance of each classifier trained on Alpha outcomes and applied to predict Omicron outcomes among our baseline models, which have not included any dynamic data, and a new set of models trained and evaluated using dynamic data. We observe a performance improvement when including dynamic data. However, there is significant missingness in the Google mobility that must be accounted for via imputation prior to training, thus complicating the model training process and reducing the practicality of our proposed method. Further, the cost associated with collecting such data on the onset of a newly emerging viral disease or variant of existing disease may be prohibitive of the approach to include dynamic data.

### Results of feature importance analysis

We report the five features that contribute the largest hold-out AUROC scores on average across the five stratified, validation folds of US counties when trained on Alpha outcomes in Table 4. From the results of Table 4, population density at the county level had the largest contribution to hold-out validation AUROC scores. During the Alpha variant period, SARS-CoV-2 immunity was low in the US; as a result, the population density of a county could be seen as an important factor for a county to experience pandemic burden. For example, a county with a higher population density may be more likely to experience pandemic burden because infectious disease, in the absence of immunity, is more likely to spread through a population dense area when compared with a population sparse (i.e., lower population density) county.

Our feature importance results further indicate that two of the top five features based on average hold-out AUROC performance are related to precipitation– April and September 2019 precipitation. We speculate that the reason our models identified an association between precipitation and Alpha outcomes is that precipitation levels are geographically clustered. That is, there are distinct clusters of counties in close geographical proximity which experience similar levels of precipitation. The inclusion of these precipitation values at the county level likely helped the ML models cluster counties in close geographic proximity and thus lead to improved discrimination scores.

**Table 4:** The five features that achieved the highest average contribution to our models’ training performance across the five-fold cross validation procedure

Feature	Contribution to AUROC
Population Density (Per Sq. Mile)	0.156
Percent of Persons (Under 19 Years) Without Health Insurance	0.013
2019 September Precipitation (in.)	0.008
2019 April Precipitation (in.)	0.005
% Population 25 Years and Over: Some College	0.005

Since our feature importance results are averaged across five stratified training folds, and many features are selected sparingly, the average contribution of these features is relatively low. In each of the five folds, Population Density was the feature that our SFLR models selected first due to its contribution to the training AUROC metric. SFLR models trained using these features averaged a validation AUROC of 0.598 (see the Omicron AUROC cell for the SFLR model in Table 3). We collectively denote these features as a parsimonious feature set and propose that they may yield some insight about the patterns of Alpha variant incidence observed in the US

## Conclusions

At the emergence of novel viruses (and their emerging variants), critical resources like health care personnel [76], ventilators [15, 69, 72], testing kits [8, 44, 59], and antiviral medication [24] may not be distributed equitably (e.g., in New York City [69]), so advance predictions of county-level incidence may help reduce outcome disparities by prioritizing resource allocation to counties likely to have the greatest need. Incidence of recent emerging variants of SARS-CoV-2, such as the Omicron variant, that led to surges in incidence and healthcare utilization rates, demonstrated wide variation among counties. Moreover, county-level outcomes varied considerably from one variant to the next. This pattern justifies the need for a data-driven algorithm to prospectively predict outcomes prior to the emergence of a new variant but recognize the challenges in doing so. In this study, we develop one such approach that leverages baseline data reported by the US Census Bureau [11] and others [see, e.g., 18, 60, 33, 39, 37] to prospectively predict outcomes of an emerging variant prior to its arrival with performance that exceeds that of plausible decision rules that decision makers might use in the absence of ML models.

We trained ML algorithms on county-level outcomes from the Alpha variant wave of SARS-CoV-2 virus in the United States. We further prospectively validated our algorithms on outcomes of the Omicron variant. Our findings show that machine learning algorithms predicted impact of future variants using data from past variants with better performance than natural heuristics that decision makers might otherwise use. Moreover, we identified a set of persistent features (publicly available prior to the US COVID-19 pandemic) that can predict SARS-CoV-2 observed incidence. Our model trained prior to outbreak of the Omicron variant could have been used by public health decision makers to identify the counties with a high need for scarce resources to combat the burden of emerging variants.

Based on our study, we speculate that there will be the continued utility of county-level classifiers as future variants of the SARS-CoV-2 virus are hypothesized to appear. Furthermore, the approaches we laid out could be applied in the event of the next pandemic generated by a novel virus. One example of a formal decision support tool, where our models could be applied, is the spatial decision support system (SDSS), which have been conceptualized by Kelly et al. [38] and then developed by Wangdi et al. [71] in the context of malaria elimination. Our predictive approach could be used in an SDSS, designed for implementation for the next pandemic, by illustrating the distribution of pandemic burden risk and enabling decision makers to strategically intervene in geographic areas with the objective to proactively contain emerging variant spread.

To our best knowledge, ours is the first validated study to predict pandemic burden associated with emerging variants of SARS-CoV-2 using ML methods applied to publicly accessible data at the county-level and across the entire US. As a result, we provide a novel tool for public health decision makers who would benefit from data-driven estimates of pandemic burden, at the county-level, for new SARS-CoV-2 variants. Furthermore, our analysis adds to the current understanding about which county-level characteristics are associated with significant risk resulting from the burden of pandemic disease [34, 49]. We hypothesize that these characteristics can be useful in characterizing the heterogeneity among outcomes within US HHS regions and not that there are causal links between county-level characteristics and pandemic outcomes. Finally, some of our findings may be applicable to early outbreak scenarios for other novel viruses, including those with emerging variants, with similar disease transmission characteristics to that of SARS-CoV-2.

### Future research

In this work, we used time-series data collected by JHU CSSE as a measure of the community spread of COVID-19. However, the data collected represents the number of confirmed cases and not the true number of cases, which would need to include asymptomatic cases and other unconfirmed diagnoses. Nevertheless, this data is the best available measure of community impact of COVID-19. Our results predict future incidence of SARS-CoV-2 cases, but this alone is not the only factor meriting consideration when making resource allocation decisions. Still, it is an important consideration for decision makers. The models obtained from our analysis have modest predictive performance compared to other use cases in the literature. Nevertheless, we have shown, they do provide significant improvements over other plausible heuristics. Future studies could examine alternative outcome metric definitions, and additional factors that might help increase the predictive performance of ML models. Our study provides an initial foundation for these future studies which may also include decision-making models such as epidemic control policies since such models were not the objective of our work. One such future study might consider the scenario where variants of a future disease appear in periods less than 100 days in length as its results may complement our work where we test outcome definitions of 100 days to align with a US White House policy document’s goals. Lastly, we caution the reader to not interpret these results as causal; namely, the relationships between the parsimonious set of predictors and the emerging variant outcomes at the county-level are not causal but instead associative – which is a promising starting point for future causal mechanism research in this context.

## Supplementary Information

This article has accompanying supplemental material.

## Supplementary Information

Below is the link to the electronic supplementary material.

Supplementary file 1 (pdf 221 KB)

## Data Availability

Data and analysis files for this study can be found at: https://github.com/kvbsmith/county-level-analysis. This individual, county-level data is available along with all codes required to generate the results in this article. This data is immediately available to anyone with access to Github for any purpose.
