# Supplementary material for: Can past variants of SARS-CoV-2 predict the impact of future variants? Machine learning for early warning of US counties at risk
Source: Health Care Manag Sci. 2025 Oct 13;28(4):738–58. doi: 10.1007/s10729-025-09728-4 (PMC12743673; doi:10.1007/s10729-025-09728-4)
Supplement: Supplementary file 1 — (pdf 221 KB) [file 10729_2025_9728_MOESM1_ESM.pdf]

Supplemental Online Content

Smith KB, Shen S, and Denton BT. Can Past Variants of SARS-CoV-2 Predict the Impact of Future Variants? Machine Learning for Early Warning of US Counties at Risk. Submitted to Health Care Management Science.

Correspondence:
Brian Denton, Stephen M. Pollock Collegiate Professor
Department of Industrial and Operations Engineering,
University of Michigan,
1891 IOE Building,
1205 Beal Ave,
Ann Arbor MI 48109-2011
E-mail: btdenton@umich.edu

eTable 1. Candidate independent variables, their descriptions, and their data sources for the study
eFigure 1. A depiction of the stratified 5-fold cross validation procedure
eTable 2. Analysis subset of independent variables and their respective VIF values
eTable 3. Retrospective and prospective performance results of machine learning analysis with 95% confidence intervals
eTable 4. A comparison of the prospective area under the receiver operating characteristic curve (AUROC) value without (i.e., standard) and with (i.e., optimized) hyperparameter optimization
eTable 5. Model performance comparison across 50 repetitions of a 5-fold cross validation between baseline heuristics and data-driven models that we report in the study and those with oversampling and class weight imbalance corrections
eTable 6. Percent of Alpha variant dynamic data missing among 3140 US counties
eTable 7. Percent of Omicron variant dynamic data missing among 3140 US counties

This supplemental material has been provided by the authors to give readers additional information about their work.

eTable 1: Candidate independent variables, their descriptions, and data sources

Independent Variable Name	Independent Variable Description	Data Source
SE_A00002_001	Total Population	ACS 5-year estimates 2014-2018 [1]
SE_A00002_002	Population Density (Per Sq. Mile)	ACS 5-year estimates 2014-2018 [1]
SE_A00002_003	Area (Land)	ACS 5-year estimates 2014-2018 [1]
PCT_SE_A02001_002	% Total Population: Male	ACS 5-year estimates 2014-2018 [1]
PCT_SE_A02001_003	% Total Population: Female	ACS 5-year estimates 2014-2018 [1]
PCT_SE_A01001_002	% Total Population: Under 5 Years	ACS 5-year estimates 2014-2018 [1]
PCT_SE_A01001_003	% Total Population: 5 to 9 Years	ACS 5-year estimates 2014-2018 [1]
PCT_SE_A01001_004	% Total Population: 10 to 14 Years	ACS 5-year estimates 2014-2018 [1]
PCT_SE_A01001_005	% Total Population: 15 to 17 Years	ACS 5-year estimates 2014-2018 [1]
PCT_SE_A01001_006	% Total Population: 18 to 24 Years	ACS 5-year estimates 2014-2018 [1]
PCT_SE_A01001_007	% Total Population: 25 to 34 Years	ACS 5-year estimates 2014-2018 [1]
PCT_SE_A01001_008	% Total Population: 35 to 44 Years	ACS 5-year estimates 2014-2018 [1]
PCT_SE_A01001_009	% Total Population: 45 to 54 Years	ACS 5-year estimates 2014-2018 [1]
PCT_SE_A01001_010	% Total Population: 55 to 64 Years	ACS 5-year estimates 2014-2018 [1]
PCT_SE_A01001_011	% Total Population: 65 to 74 Years	ACS 5-year estimates 2014-2018 [1]
PCT_SE_A01001_012	% Total Population: 75 to 84 Years	ACS 5-year estimates 2014-2018 [1]
PCT_SE_A01001_013	% Total Population: 85 Years and Over	ACS 5-year estimates 2014-2018 [1]
PCT_SE_A03001_002	% Total Population: White Alone	ACS 5-year estimates 2014-2018 [1]
PCT_SE_A03001_003	% Total Population: Black or African American Alone	ACS 5-year estimates 2014-2018 [1]
PCT_SE_A03001_004	% Total Population: American Indian and Alaska Native Alone	ACS 5-year estimates 2014-2018 [1]
PCT_SE_A03001_005	% Total Population: Asian Alone	ACS 5-year estimates 2014-2018 [1]
PCT_SE_A03001_006	% Total Population: Native Hawaiian and Other Pacific Islander Alone	ACS 5-year estimates 2014-2018 [1]
PCT_SE_A03001_007	% Total Population: Some Other Race Alone	ACS 5-year estimates 2014-2018 [1]
PCT_SE_A03001_008	% Total Population: Two or More Races	ACS 5-year estimates 2014-2018 [1]
SE_A10008_001	Households	ACS 5-year estimates 2014-2018 [1]
PCT_SE_A10008_002	% Households: Family Households	ACS 5-year estimates 2014-2018 [1]
PCT_SE_A10008_003	% Households: Family Households: Married-Couple Family	ACS 5-year estimates 2014-2018 [1]
PCT_SE_A10008_004	% Households: Family Households: Other Family	ACS 5-year estimates 2014-2018 [1]
PCT_SE_A10008_005	% Households: Family Households: Other Family: Male Householder, No Wife Present	ACS 5-year estimates 2014-2018 [1]
PCT_SE_A10008_006	% Households: Family Households: Other Family: Female Householder, No Husband Present	ACS 5-year estimates 2014-2018 [1]
PCT_SE_A10008_007	% Households: Nonfamily Households	ACS 5-year estimates 2014-2018 [1]
PCT_SE_A10008_008	% Households: Nonfamily Households: Male Householder	ACS 5-year estimates 2014-2018 [1]
PCT_SE_A10008_009	% Households: Nonfamily Households: Female Householder	ACS 5-year estimates 2014-2018 [1]
PCT_SE_A10002B_002	% Renter-Occupied Housing Units: 1-Person Household	ACS 5-year estimates 2014-2018 [1]
PCT_SE_A10002B_003	% Renter-Occupied Housing Units: 2-Person Household	ACS 5-year estimates 2014-2018 [1]
PCT_SE_A10002B_004	% Renter-Occupied Housing Units: 3-Person Household	ACS 5-year estimates 2014-2018 [1]
PCT_SE_A10002B_005	% Renter-Occupied Housing Units: 4-Person Household	ACS 5-year estimates 2014-2018 [1]
PCT_SE_A10002B_006	% Renter-Occupied Housing Units: 5-Person Household	ACS 5-year estimates 2014-2018 [1]
PCT_SE_A10002B_007	% Renter-Occupied Housing Units: 6-Person Household	ACS 5-year estimates 2014-2018 [1]
PCT_SE_A10002B_008	% Renter-Occupied Housing Units: 7-or-More Person Household	ACS 5-year estimates 2014-2018 [1]
SE_A10003_001	Average Household Size	ACS 5-year estimates 2014-2018 [1]
PCT_SE_A12001_002	% Population 25 Years and Over: Less than High School	ACS 5-year estimates 2014-2018 [1]
PCT_SE_A12001_003	% Population 25 Years and Over: High School Graduate (Includes Equivalency)	ACS 5-year estimates 2014-2018 [1]
PCT_SE_A12001_004	% Population 25 Years and Over: Some College	ACS 5-year estimates 2014-2018 [1]
PCT_SE_A12001_005	% Population 25 Years and Over: Bachelor's Degree	ACS 5-year estimates 2014-2018 [1]
PCT_SE_A12001_006	% Population 25 Years and Over: Master's Degree	ACS 5-year estimates 2014-2018 [1]
PCT_SE_A12001_007	% Population 25 Years and Over: Professional School Degree	ACS 5-year estimates 2014-2018 [1]
PCT_SE_A12001_008	% Population 25 Years and Over: Doctorate Degree	ACS 5-year estimates 2014-2018 [1]
SE_A14006_001	Median Household Income (In 2018 Inflation Adjusted Dollars)	ACS 5-year estimates 2014-2018 [1]
SE_A14024_001	Per Capita Income (In 2018 Inflation Adjusted Dollars)	ACS 5-year estimates 2014-2018 [1]
SE_A18009_001	Median Gross Rent	ACS 5-year estimates 2014-2018 [1]

Continued on next page

Continued from previous page

Independent Variable Name	Independent Variable Description	Data Source
PCT_SE_A09005_002	% Workers 16 Years and Over: Car, Truck, or Van	ACS 5-year estimates 2014-2018 [1]
PCT_SE_A09005_009	% Workers 16 Years and Over: Drove Alone	ACS 5-year estimates 2014-2018 [1]
PCT_SE_A09005_010	% Workers 16 Years and Over: Carpooled	ACS 5-year estimates 2014-2018 [1]
PCT_SE_A09005_003	% Workers 16 Years and Over: Public Transportation (Includes Taxicab)	ACS 5-year estimates 2014-2018 [1]
PCT_SE_A09005_004	% Workers 16 Years and Over: Motorcycle	ACS 5-year estimates 2014-2018 [1]
PCT_SE_A09005_005	% Workers 16 Years and Over: Bicycle	ACS 5-year estimates 2014-2018 [1]
PCT_SE_A09005_006	% Workers 16 Years and Over: Walked	ACS 5-year estimates 2014-2018 [1]
PCT_SE_A09005_007	% Workers 16 Years and Over: Other Means	ACS 5-year estimates 2014-2018 [1]
PCT_SE_A09005_008	% Workers 16 Years and Over: Worked At Home	ACS 5-year estimates 2014-2018 [1]
PCT_SE_A09001_002	% Workers 16 Years and Over: Did Not Work At Home	ACS 5-year estimates 2014-2018 [1]
PCT_SE_A09001_003	% Workers 16 Years and Over: Did Not Work At Home: Less than 10 Minutes	ACS 5-year estimates 2014-2018 [1]
PCT_SE_A09001_004	% Workers 16 Years and Over: Did Not Work At Home: 10 to 19 Minutes	ACS 5-year estimates 2014-2018 [1]
PCT_SE_A09001_005	% Workers 16 Years and Over: Did Not Work At Home: 20 to 29 Minutes	ACS 5-year estimates 2014-2018 [1]
PCT_SE_A09001_006	% Workers 16 Years and Over: Did Not Work At Home: 30 to 39 Minutes	ACS 5-year estimates 2014-2018 [1]
PCT_SE_A09001_007	% Workers 16 Years and Over: Did Not Work At Home: 40 to 59 Minutes	ACS 5-year estimates 2014-2018 [1]
PCT_SE_A09001_008	% Workers 16 Years and Over: Did Not Work At Home: 60 to 89 Minutes	ACS 5-year estimates 2014-2018 [1]
PCT_SE_A09001_009	% Workers 16 Years and Over: Did Not Work At Home: 90 or More Minutes	ACS 5-year estimates 2014-2018 [1]
PCT_SE_A09001_010	% Workers 16 Years and Over: Worked At Home	ACS 5-year estimates 2014-2018 [1]
SE_T001_001	Physically Unhealthy Days per Month (Persons 18 Years and Over)	Social Explorer Health Data 2020 [2]
SE_T001_002	Mentally Unhealthy Days per Month (Persons 18 Years and Over)	Social Explorer HD2020 [2]
SE_T006_001	Percent of Persons Without Insurance (Population Under 19 Years, 2013 est.)	Social Explorer HD2020 [2]
SE_T006_003	Percent of Persons Without Insurance (Population Under 65 Years, 2013 est.)	Social Explorer HD2020 [2]
SE_NV006_003	Premature Age-adjusted Mortality Rate per 100,000 Population	Social Explorer HD2020 [2]
SE_T009_001	Percent Diabetics (Adults)	Social Explorer HD2020 [2]
SE_T011_001	Percent Current Smokers (Persons 18 Years and Over)	Social Explorer HD2020 [2]
SE_T011_002	Percent Drinking Adults (Persons 18 Years and Over)	Social Explorer HD2020 [2]
SE_T012_001	Percent of Persons with Limited Access to Healthy Foods	Social Explorer HD2020 [2]
SE_T012_002	Percent of Persons with Access to Exercise Opportunities	Social Explorer HD2020 [2]
SE_T012_003	Percent Obese Persons (20 Years and Over)	Social Explorer HD2020 [2]
SE_T012_004	Percent Physically Inactive Persons (20 Years and Over)	Social Explorer HD2020 [2]
SE_T012_005	Percent of Children Eligible for Free Lunch (Persons $<$ 18 Years)	Social Explorer HD2020 [2]
SE_T013_001	Food Environment Index	Social Explorer HD2020 [2]
SE_T016_001	Air Pollution Particulate Matter Average Daily PM2.5	Social Explorer HD2020 [2]
SE_T016_002	Presence of Drinking Water Violations	Social Explorer HD2020 [2]
SE_T016_003	Percentage of Households with Severe Housing Problems	Social Explorer HD2020 [2]
SE_T016_004	Percentage of Households with High Housing Costs	Social Explorer HD2020 [2]
SE_T016_005	Percentage of Households with Overcrowding	Social Explorer HD2020 [2]
SE_T016_006	Percentage of Households with Lack of Kitchen or Plumbing Facilities	Social Explorer HD2020 [2]
SE_T016_007	Percent of Driving Alone to Work	Social Explorer HD2020 [2]
SE_T016_009	Percent of Long Commute Driving Alone Workers Who Drive Alone	Social Explorer HD2020 [2]
min_dist	Minimum Distance to an Airport with Scheduled Service	HumData OurAirports USA [3]
RPL_THEME1	Socioeconomic	CDC Social Vulnerability Index [4]
RPL_THEME2	Household Composition & Disability	CDC Social Vulnerability Index [4]
RPL_THEME3	Minority Status & Language	CDC Social Vulnerability Index [4]
RPL_THEME4	Housing Type & Transportation	CDC Social Vulnerability Index [4]
RPL_THEMES	Overall Tract Summary Ranking Variable	CDC Social Vulnerability Index [4]
Jan.Precipitation...inch	January Precipitation (Inches)	Killeen Group US COVID-19 Data [5]
Feb.Precipitation...inch	February Precipitation (Inches)	Killeen Group US COVID-19 Data [5]
Mar.Precipitation...inch	March Precipitation (Inches)	Killeen Group US COVID-19 Data [5]
Apr.Precipitation...inch	April Precipitation (Inches)	Killeen Group US COVID-19 Data [5]
May.Precipitation...inch	May Precipitation (Inches)	Killeen Group US COVID-19 Data [5]
Jun.Precipitation...inch	June Precipitation (Inches)	Killeen Group US COVID-19 Data [5]

Continued on next page

Continued from previous page

Independent Variable Name	Independent Variable Description	Data Source
Jul.Precipitation...inch	July Precipitation (Inches)	Killeen Group US COVID-19 Data [5]
Aug.Precipitation...inch	August Precipitation (Inches)	Killeen Group US COVID-19 Data [5]
Sep.Precipitation...inch	September Precipitation (Inches)	Killeen Group US COVID-19 Data [5]
Oct.Precipitation...inch	October Precipitation (Inches)	Killeen Group US COVID-19 Data [5]
Dec.Precipitation...inch	December Precipitation (Inches)	Killeen Group US COVID-19 Data [5]
Jan.Temp.AVG...F	January Temperature AVG (Fahrenheit)	Killeen Group US COVID-19 Data [5]
Feb.Temp.AVG...F	February Temperature AVG (Fahrenheit)	Killeen Group US COVID-19 Data [5]
Mar.Temp.AVG...F	March Temperature AVG (Fahrenheit)	Killeen Group US COVID-19 Data [5]
Apr.Temp.AVG...F	April Temperature AVG (Fahrenheit)	Killeen Group US COVID-19 Data [5]
May.Temp.AVG...F	May Temperature AVG (Fahrenheit)	Killeen Group US COVID-19 Data [5]
Jun.Temp.AVG...F	June Temperature AVG (Fahrenheit)	Killeen Group US COVID-19 Data [5]
Jul.Temp.AVG...F	July Temperature AVG (Fahrenheit)	Killeen Group US COVID-19 Data [5]
Aug.Temp.AVG...F	August Temperature AVG (Fahrenheit)	Killeen Group US COVID-19 Data [5]
Sep.Temp.AVG...F	September Temperature AVG (Fahrenheit)	Killeen Group US COVID-19 Data [5]
Oct.Temp.AVG...F	October Temperature AVG (Fahrenheit)	Killeen Group US COVID-19 Data [5]
Nov.Temp.AVG...F	November Temperature AVG (Fahrenheit)	Killeen Group US COVID-19 Data [5]
Dec.Temp.AVG...F	December Temperature AVG (Fahrenheit)	Killeen Group US COVID-19 Data [5]
f1467517	Primary Care Physicians, No. per 10,000 Population	HRSA Area Health Resource Files [6]
f0892117	Hospital Beds, No. per 1,000 Population	HRSA Area Health Resource Files [6]
life_exp_col	Life Expectancy	CDC USALEEP [7]
EP_POV	Poverty Rate, % Under Federal Poverty Level	CDC Social Vulnerability Index [4]
EP_UNEMP	Unemployment Rate	CDC Social Vulnerability Index [4]
EP_PCI	Per Capita Income, Scaled to Multiple of Federal Poverty Level	CDC Social Vulnerability Index [4]
EP_NOHSDP	Education, % Age $\geq$25 Years With No High School Degree	CDC Social Vulnerability Index [4]
EP_AGE65	Percent Aged $\geq$65	CDC Social Vulnerability Index [4]
EP_AGE17	Percent Aged $\leq$17	CDC Social Vulnerability Index [4]
EP_SNGPNT	Single Parent Household	CDC Social Vulnerability Index [4]
EP_DISABL	People With Disability (Noninstitutionalized)	CDC Social Vulnerability Index [4]
EP_MINRTY	Any Racial/Ethnic Minority	CDC Social Vulnerability Index [4]
EP_LIMENG	Limited English Proficiency	CDC Social Vulnerability Index [4]
EP_MUNIT	Housing in Structures With $\geq$10 Units	CDC Social Vulnerability Index [4]
EP_MOBILE	Mobile Homes	CDC Social Vulnerability Index [4]
EP_CROWD	Occupied Housing Units With More People Than Rooms	CDC Social Vulnerability Index [4]
EP_NOVEH	Households Without a Vehicle	CDC Social Vulnerability Index [4]
S2701_C05_012E	Adults Without Health Insurance	ACS 5-year estimates 2014-2018 [1]
S0801_C01_009E	Workers Age $\geq$16 Years Using Public Transport to Commute	ACS 5-year estimates 2014-2018 [1]
Food_Insecurity_Rate	People Lacking Access to Adequate Food	Feeding America Meal Gap Data [8]
all_icu	Intensive Care Unit Beds, No. per 10,000 Population	KHN COVID ICU Beds Data [9]

A.1-5-fold-CV.jpg

Figure 1: A depiction of the stratified 5-fold cross-validation procedure.

eTable 2: Analysis subsets including independent variables and variance inflation factor (VIF) values

**Independent Variable**	**VIF**
(Intercept)	449.08
nov_precipitation_inch	1.93
pct_se_a09001_005	1.87
pct_se_a09001_008	1.81
apr_precipitation_inch	1.81
jul_precipitation_inch	1.80
pct_se_a09001_006	1.74
aug_precipitation_inch	1.72
mar_precipitation_inch	1.71
se_t006_001	1.71
may_precipitation_inch	1.70
min_dist	1.70
se_t012_003	1.69
pct_se_a01001_009	1.67
ep_unemp	1.66
pct_se_a12001_008	1.66
se_t009_001	1.66
se_t012_002	1.65
pct_se_a03001_008	1.64

Continued on next page

Continued from previous page

**Independent Variable**	**VIF**
pct_se_a01001_008	1.56
pct_se_a12001_004	1.56
pct_se_a01001_005	1.54
pct_se_a09001_009	1.53
se_a00002_003	1.50
pct_se_a10008_005	1.46
pct_se_a09005_005	1.44
sep_precipitation_inch	1.43
pct_se_a09005_007	1.41
se_t012_001	1.40
all_icu	1.39
pct_se_a03001_006	1.35
ep_groupq	1.34
pct_se_a03001_007	1.33
pct_se_a10002b_008	1.32
se_a00002_002	1.29
pct_se_a10002b_006	1.26
pct_se_a10002b_003	1.26
pct_se_a10002b_005	1.26
pct_se_a10002b_004	1.26
pct_se_a10002b_007	1.23
pct_se_a09005_010	1.22
pct_se_a09005_004	1.10

eTable 3: Retrospective and prospective performance results of machine learning analysis with 95% confidence intervals

Algorithm	AUROC Test	Precision (Alpha)			Precision (Omicron)			Recall (Omicron)		
		@50	@100	@500	@50	@100	@500	@50	@100	@500
SFLR	0.599 (0.597 - 0.601)	0.606 (0.600 - 0.612)	0.512 (0.507 - 0.518)	0.921 (0.919 - 0.923)	0.346 (0.341 - 0.351)	0.322 (0.319 - 0.325)	0.873 (0.871 - 0.875)	0.280 (0.274 - 0.285)	0.286 (0.282 - 0.289)	0.291 (0.291 - 0.292)
RF	0.582 (0.581 - 0.583)	0.782 (0.778 - 0.787)	0.633 (0.629 - 0.636)	0.933 (0.931 - 0.934)	0.373 (0.369 - 0.376)	0.319 (0.317 - 0.322)	0.877 (0.875 - 0.879)	0.297 (0.292 - 0.302)	0.289 (0.286 - 0.292)	0.291 (0.290 - 0.292)
SGBAP	0.598 (0.598 - 0.599)	0.642 (0.638 - 0.646)	0.526 (0.523 - 0.529)	0.907 (0.905 - 0.909)	0.348 (0.343 - 0.352)	0.314 (0.312 - 0.317)	0.877 (0.875 - 0.878)	0.302 (0.299 - 0.306)	0.288 (0.286 - 0.290)	0.288 (0.287 - 0.288)
Population Density	0.588 (0.588 - 0.588)	0.535 (0.533 - 0.537)	0.456 (0.455 - 0.457)	0.918 (0.917 - 0.919)	0.322 (0.320 - 0.324)	0.311 (0.310 - 0.312)	0.875 (0.874 - 0.875)	0.426 (0.423 - 0.428)	0.393 (0.392 - 0.394)	0.302 (0.301 - 0.302)
Alpha Cases	0.555 (0.553 - 0.557)	- -	- -	- -	0.371 (0.370 - 0.372)	0.308 (0.301 - 0.315)	0.871 (0.869 - 0.873)	0.291 (0.290 - 0.292)	0.343 (0.339 - 0.347)	0.288 (0.283 - 0.293)
SBLR	0.594 (0.593 - 0.596)	0.635 (0.629 - 0.640)	0.530 (0.527 - 0.533)	0.933 (0.931 - 0.934)	0.349 (0.345 - 0.354)	0.329 (0.326 - 0.332)	0.872 (0.870 - 0.873)	0.289 (0.285 - 0.293)	0.283 (0.280 - 0.287)	0.290 (0.289 - 0.290)

eTable 4: A comparison of the prospective area under the receiver operating characteristic curve (AUROC) value without (i.e., standard) and with (i.e., optimized) hyperparameter optimization

**Algorithm**	**Standard AUROC**	**Optimized AUROC**	**% Difference**
SFLR	0.597	0.597	0.000%
SBLR	0.594	0.595	0.168%
RF	0.586	0.589	0.512%

eTable 5: Model performance comparison across 50 repetitions of a 5-fold cross validation between baseline heuristics and data-driven models that we report in the study and those with oversampling and class weight imbalance corrections

**Algorithm**	**Imbalance Correction**	**AUROC (95% CI)**	**% Difference to baseline**
Population Density	None (baseline)	0.588 (0.588 - 0.588)	–
Alpha Cases	None (baseline)	0.555 (0.555 - 0.555)	–
SGBAP	None (baseline)	0.598 (0.597 - 0.599)	–
Random Forest	None (baseline)	0.582 (0.581 - 0.583)	–
	Oversampling	0.584 (0.583 - 0.585)	3.4%
	Class Weight	0.585 (0.583 - 0.586)	5.2%
SBLR	None (baseline)	0.594 (0.593 - 0.596)	–
	Oversampling	0.592 (0.590 - 0.593)	-3.4%
	Class Weight	0.595 (0.594 - 0.597)	1.7%
SFLR	None (baseline)	0.599 (0.597 - 0.601)	–
	Oversampling	0.602 (0.600 - 0.604)	5%
	Class Weight	0.601 (0.599 - 0.603)	3.3%

eTable 6: Percent of Alpha variant dynamic data missing among 3140 US counties.

Dynamic Feature Name	Percent Missing
val_pct_cli_week_ending_4_11_2020	56.1%
val_pct_hh_cmnty_cli_week_ending_4_11_2020	100.0%
val_pct_nohh_cmnty_cli_week_ending_4_11_2020	100.0%
val_pct_ili_week_ending_4_11_2020	56.1%
val_pct_direct_contact_week_ending_4_11_2020	63.6%
val_pct_direct_contact_covid_week_ending_4_11_2020	60.3%
val_pct_direct_contact_covid_hh_week_ending_4_11_2020	60.3%
val_pct_symp_fever_week_ending_4_11_2020	57.2%
val_pct_symp_cough_week_ending_4_11_2020	57.2%
val_pct_symp_diff_breathing_week_ending_4_11_2020	57.2%
val_pct_symp_fatigue_week_ending_4_11_2020	57.2%
val_pct_symp_aches_week_ending_4_11_2020	57.2%
val_pct_symp_sore_throat_week_ending_4_11_2020	57.2%
val_pct_symp_chest_pain_week_ending_4_11_2020	57.2%
val_pct_symp_nausea_week_ending_4_11_2020	57.2%
val_pct_symp_other_week_ending_4_11_2020	57.2%
val_pct_symp_none_week_ending_4_11_2020	57.2%
val_pct_symp_shortness_breath_week_ending_4_11_2020	57.2%
val_pct_symp_nasal_congestion_week_ending_4_11_2020	57.2%
val_pct_symp_runny_nose_week_ending_4_11_2020	57.2%
val_pct_symp_diarrhea_week_ending_4_11_2020	57.2%
val_pct_symptom_hospital_week_ending_4_11_2020	76.7%
val_pct_symptom_hospital_tried_week_ending_4_11_2020	76.7%
val_pct_work_outside_home_5d_week_ending_4_11_2020	59.4%
val_pct_work_healthcare_5d_week_ending_4_11_2020	59.3%
val_pct_work_nursing_home_5d_week_ending_4_11_2020	59.3%
val_pct_travel_outside_state_5d_week_ending_4_11_2020	59.3%
val_pct_avoid_contact_week_ending_4_11_2020	59.2%
val_pct_avoid_contact_7d_week_ending_4_11_2020	100.0%
val_mean_days_symptoms_week_ending_4_11_2020	77.9%
val_mean_ppl_symptoms_household_week_ending_4_11_2020	56.1%
val_mean_ppl_symptoms_community_week_ending_4_11_2020	100.0%
val_pct_taken_temp_week_ending_4_11_2020	99.9%
val_pct_cough_mucus_week_ending_4_11_2020	89.8%
val_pct_unusual_symptom_tested_week_ending_4_11_2020	76.8%
val_pct_unusual_symptom_tested_positive_week_ending_4_11_2020	76.8%
val_pct_financial_threat_week_ending_4_11_2020	61.0%
seven_day_ma_week_ending_3_21_2020	0.0%
seven_day_ma_week_ending_3_28_2020	0.0%
seven_day_ma_week_ending_4_04_2020	0.0%
retail_and_recreation_percent_change_from_baseline_avg_week_ending_4_04_2020	19.2%
grocery_and_pharmacy_percent_change_from_baseline_avg_week_ending_4_04_2020	21.8%
parks_percent_change_from_baseline_avg_week_ending_4_04_2020	71.9%
transit_stations_percent_change_from_baseline_avg_week_ending_4_04_2020	65.0%
workplaces_percent_change_from_baseline_avg_week_ending_4_04_2020	11.8%
residential_percent_change_from_baseline_avg_week_ending_4_04_2020	59.2%
retail_and_recreation_percent_change_from_baseline_avg_week_ending_3_28_2020	18.9%
grocery_and_pharmacy_percent_change_from_baseline_avg_week_ending_3_28_2020	21.5%
parks_percent_change_from_baseline_avg_week_ending_3_28_2020	71.9%
transit_stations_percent_change_from_baseline_avg_week_ending_3_28_2020	65.2%
workplaces_percent_change_from_baseline_avg_week_ending_3_28_2020	12.0%
residential_percent_change_from_baseline_avg_week_ending_3_28_2020	58.2%
retail_and_recreation_percent_change_from_baseline_avg_week_ending_3_21_2020	19.0%
grocery_and_pharmacy_percent_change_from_baseline_avg_week_ending_3_21_2020	22.8%
parks_percent_change_from_baseline_avg_week_ending_3_21_2020	73.2%
transit_stations_percent_change_from_baseline_avg_week_ending_3_21_2020	65.9%
workplaces_percent_change_from_baseline_avg_week_ending_3_21_2020	12.6%
residential_percent_change_from_baseline_avg_week_ending_3_21_2020	53.9%

eTable 7: Percent of Omicron variant dynamic data missing among 3140 US counties.

Dynamic Feature Name	Percent Missing
val_pct_cli_week_ending_2_12_2022	88.3%
val_pct_hh_cmnty_cli_week_ending_2_12_2022	88.9%
val_pct_nohh_cmnty_cli_week_ending_2_12_2022	88.9%
val_pct_ili_week_ending_2_12_2022	88.3%
val_pct_direct_contact_week_ending_2_12_2022	100.0%
val_pct_direct_contact_covid_week_ending_2_12_2022	100.0%
val_pct_direct_contact_covid_hh_week_ending_2_12_2022	100.0%
val_pct_symp_fever_week_ending_2_12_2022	88.7%
val_pct_symp_cough_week_ending_2_12_2022	88.7%
val_pct_symp_diff_breathing_week_ending_2_12_2022	88.7%
val_pct_symp_fatigue_week_ending_2_12_2022	88.7%
val_pct_symp_aches_week_ending_2_12_2022	88.7%
val_pct_symp_sore_throat_week_ending_2_12_2022	88.7%
val_pct_symp_chest_pain_week_ending_2_12_2022	88.7%
val_pct_symp_nausea_week_ending_2_12_2022	88.7%
val_pct_symp_other_week_ending_2_12_2022	88.7%
val_pct_symp_none_week_ending_2_12_2022	88.7%
val_pct_symp_shortness_breath_week_ending_2_12_2022	88.7%
val_pct_symp_nasal_congestion_week_ending_2_12_2022	100.0%
val_pct_symp_runny_nose_week_ending_2_12_2022	100.0%
val_pct_symp_diarrhea_week_ending_2_12_2022	88.7%
val_pct_symptom_hospital_week_ending_2_12_2022	100.0%
val_pct_symptom_hospital_tried_week_ending_2_12_2022	100.0%
val_pct_work_outside_home_5d_week_ending_2_12_2022	100.0%
val_pct_work_healthcare_5d_week_ending_2_12_2022	100.0%
val_pct_work_nursing_home_5d_week_ending_2_12_2022	100.0%
val_pct_travel_outside_state_5d_week_ending_2_12_2022	100.0%
val_pct_avoid_contact_week_ending_2_12_2022	100.0%
val_pct_avoid_contact_7d_week_ending_2_12_2022	89.0%
val_mean_days_symptoms_week_ending_2_12_2022	99.6%
val_mean_ppl_symptoms_household_week_ending_2_12_2022	88.3%
val_mean_ppl_symptoms_community_week_ending_2_12_2022	88.9%
val_pct_taken_temp_week_ending_2_12_2022	100.0%
val_pct_cough_mucus_week_ending_2_12_2022	100.0%
val_pct_unusual_symptom_tested_week_ending_2_12_2022	100.0%
val_pct_unusual_symptom_tested_positive_week_ending_2_12_2022	100.0%
val_pct_financial_threat_week_ending_2_12_2022	100.0%
seven_day_ma_week_ending_1_29_2022	0.0%
seven_day_ma_week_ending_2_05_2022	0.0%
seven_day_ma_week_ending_2_12_2022	0.0%
retail_and_recreation_percent_change_from_baseline_avg_week_ending_2_12_2022	43.5%
grocery_and_pharmacy_percent_change_from_baseline_avg_week_ending_2_12_2022	48.6%
parks_percent_change_from_baseline_avg_week_ending_2_12_2022	73.7%
transit_stations_percent_change_from_baseline_avg_week_ending_2_12_2022	66.7%
workplaces_percent_change_from_baseline_avg_week_ending_2_12_2022	13.2%
residential_percent_change_from_baseline_avg_week_ending_2_12_2022	43.3%
retail_and_recreation_percent_change_from_baseline_avg_week_ending_2_05_2022	43.0%
grocery_and_pharmacy_percent_change_from_baseline_avg_week_ending_2_05_2022	48.2%
parks_percent_change_from_baseline_avg_week_ending_2_05_2022	72.4%
transit_stations_percent_change_from_baseline_avg_week_ending_2_05_2022	65.8%
workplaces_percent_change_from_baseline_avg_week_ending_2_05_2022	12.5%
residential_percent_change_from_baseline_avg_week_ending_2_05_2022	43.6%
retail_and_recreation_percent_change_from_baseline_avg_week_ending_1_29_2022	43.1%
grocery_and_pharmacy_percent_change_from_baseline_avg_week_ending_1_29_2022	48.4%
parks_percent_change_from_baseline_avg_week_ending_1_29_2022	72.8%
transit_stations_percent_change_from_baseline_avg_week_ending_1_29_2022	66.4%
workplaces_percent_change_from_baseline_avg_week_ending_1_29_2022	13.1%
residential_percent_change_from_baseline_avg_week_ending_1_29_2022	44.0%

References

[1] US Census Bureau. American community survey 2014-2018 5-year estimates, (accessed April 14, 2021).

[2] County Health Rankings & Roadmaps. Health data 2020 release, (accessed April 13, 2021).

[3] United Nations Office for the Coordination of Humanitarian Affairs (OCHA). Airports in the United States of America, (accessed April 30, 2021).

[4] Centers for Disease Control and Prevention/ Agency for Toxic Substances and Disease Registry/ Geospatial Research, Analysis, and Services Program. Cdc/atsdr social vulnerability index 2018 us counties, (accessed July 1, 2021).

[5] Benjamin D Killeen, Jie Ying Wu, Kinjal Shah, Anna Zapaishchykova, Philipp Nikutta, Aniruddha Tamhane, Shreya Chakraborty, Jinchi Wei, Tiger Gao, Mareike Thies, et al. A county-level dataset for informing the united states' response to covid-19. *arXiv preprint arXiv:2004.00756*, 2020.

[6] U.S. Department of Health, Human Services Health Resources, and Services Administration. Area health resources file. 2018., (accessed July 4, 2021).

[7] National Center for Health Statistics. U.s. small-area life expectancy estimates project (usaleep): Life expectancy estimates file for Jurisdiction, 2010-2015, (accessed June 25, 2021).

[8] C. Gundersen, A. Dewey, E. Engelhard, M. Strayer, and L Lapinski. Map the meal gap 2020: A report on county and congressional district food insecurity and county food cost in the united states in 2018., (accessed June 24, 2021).

[9] Kaiser Health News. Icu beds by county data, (accessed June 22, 2021).
